# Lineage specific extracellular vesicle-associated protein biomarkers for the early detection of high grade serous ovarian cancer

**DOI:** 10.1038/s41598-023-44050-5

**Published:** 2023-10-26

**Authors:** Camille V. Trinidad, Harsh B. Pathak, Shibo Cheng, Shin-Cheng Tzeng, Rashna Madan, Mihaela E. Sardiu, Leonidas E. Bantis, Clayton Deighan, Andrea Jewell, Sagar Rayamajhi, Yong Zeng, Andrew K. Godwin

**Affiliations:** 1 Department of Microbiology, Molecular Genetics and Immunology, University of Kansas Medical Center, Kansas City, KS USA; 2 Department of Pathology and Laboratory Medicine, The University of Kansas Medical Center, 3901 Rainbow Boulevard, MS 3040, Kansas City, KS 66160 USA; 3 University of Kansas Cancer Center, Kansas City, KS USA; 4 Kansas Institute for Precision Medicine, University of Kansas Medical Center, Kansas City, KS USA; 5 Department of Chemistry, University of Florida, Gainesville, FL USA; 6 The Donald Danforth Plant Science Center, St. Louis, MO USA; 7 Department of Biostatistics and Data Science, University of Kansas Medical Center, Kansas City, KS USA; 8 NanoFCM, Nottingham, Nottinghamshire, UK; 9 Department of Obstetrics and Gynecology, University of Kansas Medical Center, Kansas City, KS USA

**Keywords:** Cancer, Cancer, Tumour biomarkers, Biomarkers

## Abstract

High grade serous ovarian carcinoma (HGSOC) accounts for ~ 70% of ovarian cancer cases. Non-invasive, highly specific blood-based tests for pre-symptomatic screening in women are crucial to reducing the mortality associated with this disease. Since most HGSOCs typically arise from the fallopian tubes (FT), our biomarker search focused on proteins found on the surface of extracellular vesicles (EVs) released by both FT and HGSOC tissue explants and representative cell lines. Using mass spectrometry, 985 EV proteins (exo-proteins) were identified that comprised the FT/HGSOC EV core proteome. Transmembrane exo-proteins were prioritized because these could serve as antigens for capture and/or detection. With a nano-engineered microfluidic platform, six newly discovered exo-proteins (ACSL4, IGSF8, ITGA2, ITGA5, ITGB3, MYOF) plus a known HGSOC associated protein, FOLR1 exhibited classification performance ranging from 85 to 98% in a case–control study using plasma samples representative of early (including stage IA/B) and late stage (stage III) HGSOCs. Furthermore, by a linear combination of IGSF8 and ITGA5 based on logistic regression analysis, we achieved a sensitivity of 80% with 99.8% specificity and a positive predictive value of 13.8%. Importantly, these exo-proteins also can accurately discriminate between ovarian and 12 types of cancers commonly diagnosed in women. Our studies demonstrate that these lineage-associated exo-biomarkers can detect ovarian cancer with high specificity and sensitivity early and potentially while localized to the FT when patient outcomes are more favorable.

## Introduction

Epithelial ovarian cancer is associated with an overall mortality of ~ 70% but can be cured in up to 90% of cases if diagnosed early while still restricted to the ovaries and fallopian tubes^1–4^. Unfortunately, most cases, including the most aggressive and deadly form of ovarian cancer^5^, *i.e.,* high grade serous ovarian cancer (HGSOC), are diagnosed in the late stages, when the five-year survival rate falls below 30%^4^. Disappointingly, only about 15% of the cases are localized to the reproductive tissues at the time of diagnosis^6^. Therefore, only a few cases are cured. The lack of precise early warning signs is one of the contributing factors to the small percentage of detection of ovarian tumors in stage I/II^7–9^. EOC screening requires surgical methods for a confirmatory diagnosis, in contrast to breast cancer where a biopsy can be used to diagnose the disease^6,10^. Given the low prevalence of this cancer in the postmenopausal women (~ 1 in 2500) effective screening strategies are needed to achieve a positive predictive value (PPV) higher than 10% (one positive surgery out of 10)^10–13^.

The two most used ovarian screening tests in the clinic are transvaginal ultrasound (TVS) and a blood test quantifying the serum level of CA-125, which is often not reliable given elevated CA-125 levels are also caused by non-malignant gynecological conditions^14,15^. When a mass is found with TVS, a more invasive biopsy is required. Ideally, a non-invasive route for diagnosis is preferred, but there is currently no consensus on a blood-based biomarker panel for the early detection of this disease.

The UK Collaborative Trial of Ovarian Cancer Screening (UKCTOCS) has led the way to determine if population screening can reduce deaths due to the disease with over 200,000 postmenopausal women recruited in this study. When the risk of ovarian cancer algorithm (ROCA) was combined with TVS for women who had elevated CA-125 (a multimodal screening approach), this approach resulted in a combined specificity of 99.8% with a 22% PPV and a modest increase in the detected early stage EOC cases^10,16^. However, after 16.3 years of long-term follow-up this screening trial did not yield promising results because the mortality rates were not significantly reduced even with screening^17^. This clinical problem underscores the importance of identifying blood-based biomarkers for detecting cancers early when the disease is more manageable.

To further complicate the screening for ovarian cancer, recent studies continue to support the hypothesis that most HGSOCs arise from the fallopian tube (FT) epithelium, specifically the secretory cells in the fimbriated end^18–20^. The development of the earliest steps in the pathogenesis of HGSOC is thought to take decades; however, recent molecular studies have suggested that the transition from a serous tubal intraepithelial carcinoma (STIC) into a metastatic ovarian carcinoma occurs much more rapid; between 6 and 7 years^18,21^. Once the tumor cell leaves the fallopian tube the disease spreads rapidly and is hard to cure with existing therapies. In fact, one might contemplate renaming this “ovarian cancer” subtype given the tissue of origin. If this biology is indeed correct, we hypothesized that identifying novel FT-associated protein biomarkers during STIC development would provide clinicians a window of opportunity to diagnose the disease at an earlier stage and to surgically remove one or both fallopian tubes (salpingectomy) to reduce the chance of metastasis. There is new evidence that opportunistic salpingectomy in patients already undergoing pelvic surgery for benign disease such as a hysterectomy may significantly decrease ovarian cancer risk^22,23^ which the Society of Gynecologic Oncology has recommended. Also, the SOROCk (Salpingo-Oophorectomy to Reduce the risk of Ovarian Cancer—NCT04251052) clinical trial is currently recruiting women to determine the efficacy of bilateral salpingectomy with delayed oophorectomy in high risk women for ovarian cancer prevention.

To detect HGSOCs in its early forms and through non-invasive methods, we focused on novel proteins in extracellular vesicles (EVs) (exo-protein biomarkers). EVs are nanosized particles that are between 30 nm and 1 µm in diameter^24^. EVs are typically formed when vesicles bud off from the plasma membrane of live or dying cells (shedding microvesicles or apoptotic bodies, respectively) or when endosomes (late or recycling) fuse to the plasma membrane which results in the release of multivesicular bodies including small EVs (a.k.a. exosomes)^25^. EVs contain nucleic acids, lipids, and proteins that reflect the cargo of the cell of origin^26^. Many studies have shown that tissue explants are a rich source of these EVs, and that these EVs contain potential biomarkers for various diseases^27^. For the proteomic profiling studies presented in this manuscript, we use the term EVs to describe vesicles recovered in the 100,000×g centrifugation step, which are primarily 50–200 nm in size.

Several studies, including ours have used microfluidic devices to capture EVs and quantify levels of proteins such as CD24, CA-125, EpCAM, EGFR, HER2 and/or FOLR1 from ovarian cancer patients’ plasma or ascites samples^28–32^. Although these previous studies are promising, there is a still a need to uncover lineage specific biomarkers for cancers originating in the fallopian tubes to increase the sensitivity and specificity of these tests to support screening studies, especially in women with who are at an increased risk (*e.g.*, *BRCA1* and *BRCA2* mutation carriers).

To address this clinical need we performed extensive proteomic analyses by mass spectrometry of EVs enriched from healthy FT and HGSOC short-term tissue explants, as well as established fallopian tube secretory epithelial cells (FTSEC)- and HGSOC-derived cell lines. We report the identification of EV-associated proteins that have potential to serve as circulating early detection biomarkers of HGSOC that can be detected in a few drops of blood. We hypothesized that EVs from tissue explants could identify relevant biomarkers for human disease and complement those found using cultured cells, which is not exposed to other cell types in the tumor microenvironment^33^ as would the tissue explants. From these studies, we identified 7 transmembrane candidate protein markers (ACSL4, IGSF8, ITGA2, ITGA5, ITGB3, MYOF and STX4) on EVs derived from FT and HGSOC explants. We further evaluated these candidate markers in clinical samples from women diagnosed with HGSOC and healthy age/race matched controls using a novel nano-engineered microfluidic platform (ExoProfile chip^32^) and showed that these markers exhibit high area under the curve (AUC) values ranging from 0.85 to 0.98 as calculated using receiver operating characteristic (ROC) analysis. When multi-marker analysis was performed, the combination of IGSF8 and ITGA5 yielded the greatest degree of sensitivity and specificity in this cohort.

Previous studies have focused on other types of cancer such as colorectal cancer, lung cancer, melanoma, and pancreatic cancer^27,34^. To our knowledge, this is the first study to comprehensively compare the proteomes of EVs from short-term cultures of tissue explants of primary FT and HGSOC with those from established and representative FTSEC and HGSOC cell lines. This study suggests that enrichment and characterization of EVs from short-term cultures of tissue explants maybe a valuable and complementary approach to uncover additional circulating biomarkers not observed with cultured cells. Our study highlights the importance of tissue derived EVs in biomarker discovery and may have biological implications on the disease pathogenesis of HGSOC.

## Results

### Enrichment and characterization of EVs derived from patient HGSOC and healthy fallopian tube tissue explants and cell lines

**Figure 1:**
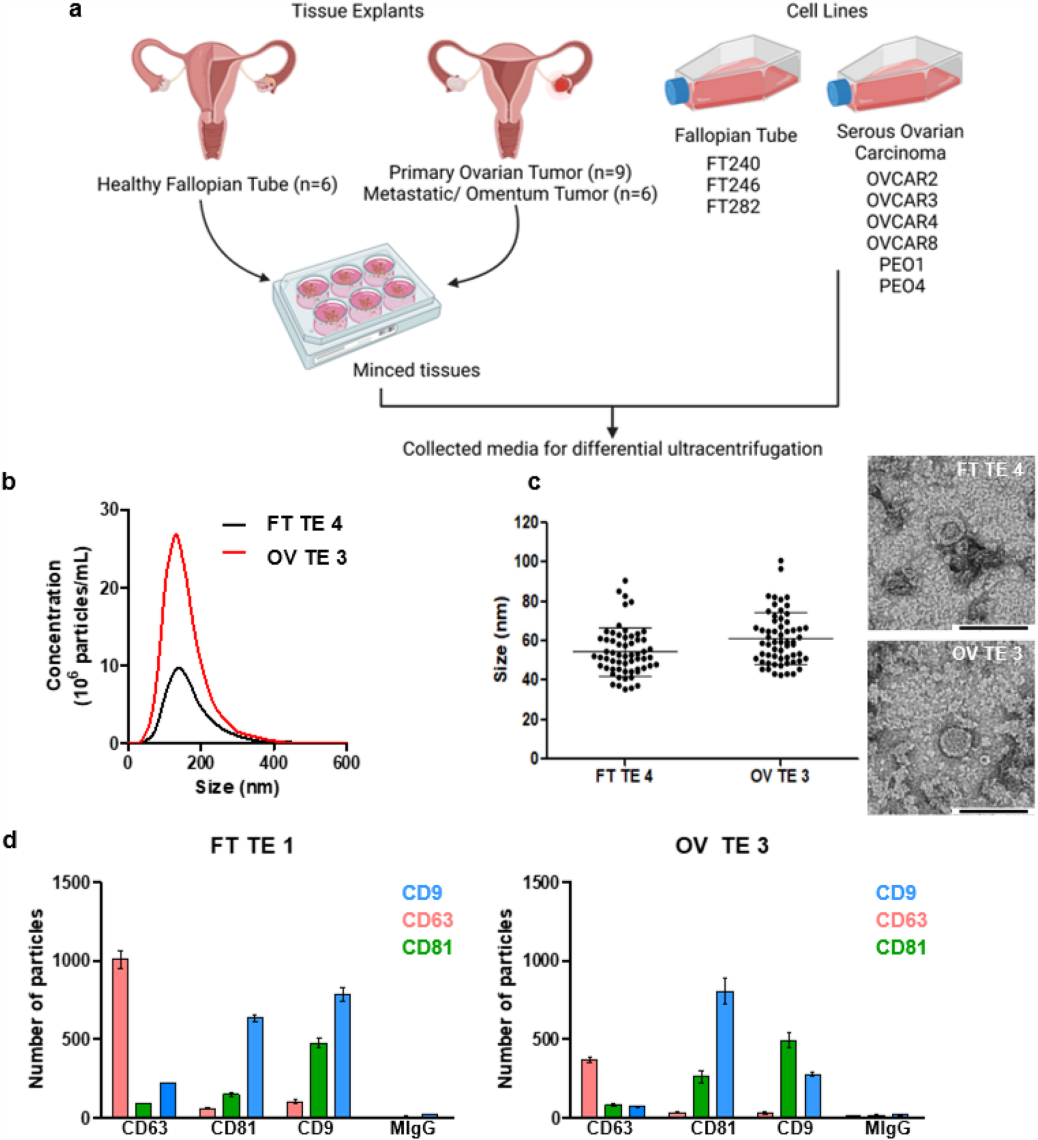
Enrichment and characterization of cell line and tissue explant EVs. (**a**) Surgical resections of healthy FT or tumor tissues were minced and used to initiate short-term tissue explants (cultured for 24 h) followed by collection of the conditioned media and processed by differential ultracentrifugation to enrich for EVs. Likewise, conditioned media from the FT and ovarian cancer cell lines shown was collected and processed. Created with BioRender.com (**b**) Representative NTA data. (**c**) Sixty (60) EV particles were imaged, and their size was measured for representative samples by TEM at x30K magnification. The mean size with s.e.m. is indicated by the error bars. (**d**) Shown are representative fluorescence data obtained using ExoView for FT and HGSOC tissue explant derived EVs. The EVs were captured using commonly expressed EV tetraspanins, namely, CD9, CD63 and CD81 and probed with detection antibodies conjugated to Alexa Fluor dyes: CD9-AF488 (blue), CD63-AF647 (pink) and CD81-AF555 (green). The error bars represent the mean particle count with s.e.m.

Conditioned media was collected from 3 FT cell lines (FT240, FT246 and FT282), 6 HGSOC cell lines (OVCAR2, OVCAR3, OVCAR4, OVCAR8, PEO1 and PEO4) and 21 fresh tissue explants (HGSOC primary tumor tissues, n = 9, HGSOC omental metastases, n = 6, and healthy FT tissue specimens, n = 6) (Fig. 1a & Supplementary File 1). For these studies, we chose the 24 h time point for tissue media collection since the number of total particles decreased by at least 55% within 48 h (Supplementary Fig. 1). This observation might be attributed to the decrease in growth factors once the media is replaced at 24 h. The collected media was then processed by differential ultracentrifugation^27^ to enrich for the EVs. We characterized representative EVs purified from either cell lines or tissue explants by nanoparticle tracking analysis (NTA), transmission electron microscopy (TEM), single particle interferometric reflectance imaging sensing (SP-IRIS) and fluorescence following the *Minimum Information for Studies of Extracellular Vesicles 2018* guidelines^35^ (Fig. 1b–d & Supplementary Fig. 2–3). By NTA, most particles were between 120 and 160 nm in size (Fig. 1b & Supplementary Fig. 3a). Using negative staining followed by TEM, we observed EVs to have the typical cup shaped morphology with a size range between 32 and 128 nm (Fig. 1c & Supplementary Fig. 3b). By SP-IRIS, the mode size for the EVs is 50 nm (Supplementary Fig. 3c). The variation in EV particle size as detected by NTA, TEM, and SP-IRIS and fluorescence is likely due to the differences in instrument sensitivities and their limitations (Fig. 1b–c & Supplementary Fig. 3a-c). With SP-IRIS and fluorescence, we found that both cell line and tissue explant EVs either from FT or HGSOC expressed common tetraspanin markers, *e.g.,* CD9, CD63 and CD81 (Fig. 1d & Supplementary Fig. 3d). We also observed that tissue explant derived EVs had a higher percentage of CD63 single positive EVs compared to cell line derived EVs (Fig. 1d) whereas FT cell lines and HGSOC cell lines displayed a higher CD9^+^ population, as well as double positive CD9^+^CD81^+^ EV populations compared to the tissue explant derived EVs (Fig. 1d, Supplementary Fig. 3d & Supplementary Fig. 4). This finding could be due to the presence of additional cell populations (*e.g.*, tumor stroma) present in the tissue explants releasing EVs or the overall differences in the biogenesis of tissue derived EVs compared to that of cell line EVs, similar to how 2D culture and 3D culture derived EVs differ in EV populations^36^.

### Identification of FT and HGSOC core proteome

**Figure 2:**
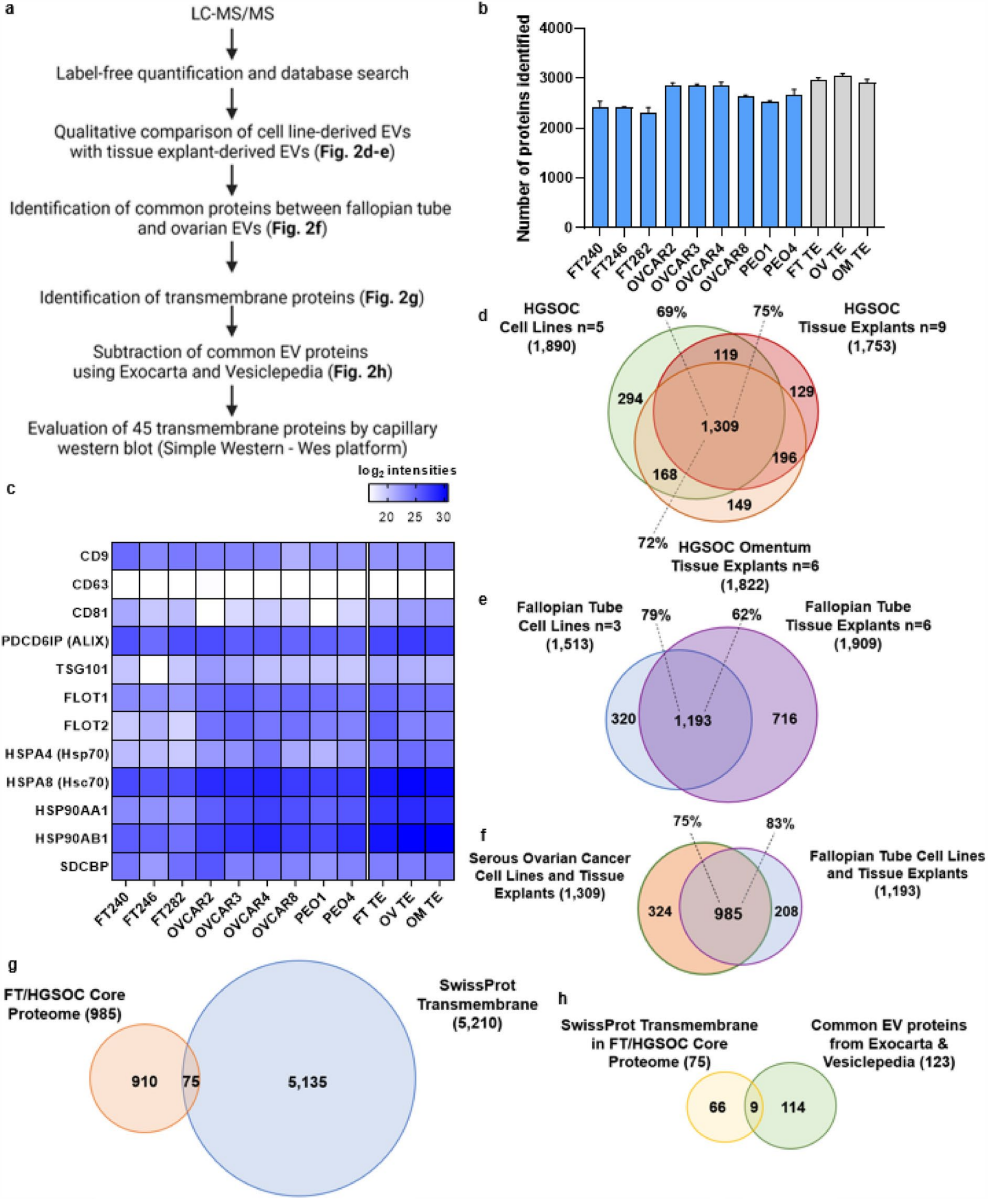
Proteomic analysis of tissue explant and cell line EVs. (**a**) Pipeline for filtering LC–MS/MS data to aide in selection of potential transmembrane candidate protein biomarkers. Created with BioRender.com. (**b**) Shown are the initial number of proteins identified in cell line EVs (blue, average of two or three EV isolations from conditioned media) and tissue explant EVs (gray, average of all samples from their respective groups). (**c**) Heatmap of proteomic data showing enrichment of common EV protein markers for both cell line and tissue derived EVs. (**d**) Venn diagram comparison of protein distribution between HGSOC cell lines and tissue explants, and (**e**) between FT cell lines and FT tissue explants. (**f**) Identification of the FT/HGSOC core proteome by comparison of common proteins between the two groups (HGSOC EVs and FT EVs). (**g**) Identification of transmembrane proteins within the FT/HGSOC core proteome by comparison to the SwissProt predicted transmembrane database, and (**h**) removal of expected/common EV proteins within the transmembrane FT/HGSOC core proteome by comparison to the Exocarta and Vesiclepedia.

After EV characterization, we performed proteomic profiling and an analysis pipeline (Fig. 2a) to establish the EV proteome of FT and HGSOC (for both tissue and cell lines) via liquid chromatography tandem mass spectrometry (LC–MS/MS)^27,34^, with the goal of identifying putative transmembrane exo-proteins that can ultimately be used to perform immunocapture and detection of intact EVs from clinical samples.

To determine which exo-proteins were to be included in subsequent analyses, a criteria was set such that a protein was included only if it was detected in at least two out of the three biological replicates for the HGSOC or in both biological replicates for the FT cell lines. For the tissue explants, the criteria required that the proteins were detected in all the biological replicates within their respective set. Using this approach, approximately 2200–3200 exo-proteins were identified in each sample (Fig. 2b). Then, we calculated the relative abundance of the common EV markers using our proteomic data and found that most of these markers were identified at similar levels in both the cell line and tissue explant derived EVs (Fig. 2c). However, a common EV marker, CD63, was detected at relatively low levels or was undetectable in EVs, as observed in previous studies^27^. Lack of representation in the mass spectrometry data is likely due to the CD63 being heavily glycosylated^37^. As mentioned earlier, we found that CD63 + EVs was predominately present in tissue explants derived EVs using SP-IRIS and fluorescence. Our findings also support a recent study that proposed syntenin-1 (SDCBP) to be a putative universal EV marker^38^ as this protein was detected at relatively similar levels across all the EV samples (both tissue and cell line-derived). In addition, we examined the relative quantitation of serum-based proteins that are reported in literature or included in biomarker-based algorithms for ovarian cancer, *i.e.*, CA-125^39^; ROCA multimodal screening^10,17^; the multivariate ROMA^40^ test; and the FDA approved OVA1^41^ test. Importantly, these serum markers were also found in the EVs used in our study (Supplementary Fig. 5).

After this initial assessment of the data quality by comparing levels of the canonical EV markers as well as presence of existing serum markers from the tests mentioned above, we filtered the data furthered by comparing the EV proteins from tissue explants with their respective cell lines (*e.g.*, HGSOC or FT) to increase the specificity of the EV proteins to their site of origin (Fig. 2d–e). We then compared the 1309 proteins found in the HGSOC group with the 1193 proteins found in the FT group to identify 985 EV proteins that are common between the two groups which we termed as FT/HGSOC core proteome (Fig. 2f & Supplementary File 2). We have performed an extensive ROC analysis of the 985 core FT/HGSOC proteome and have identified a list of 43 monotone and non-monotone markers^42^ (the definition of each marker type is provided in the Material and Methods section), including non-transmembrane and cytosolic proteins as well as a bioinformatic analysis of the 985 core proteins and 324 markers unique to HGSOC (Fig. 2f). A detailed description of these analyses is presented in Supplementary File 3.

### Identification of transmembrane proteins

The above ROC analysis is useful for identifying differences between FT and HGSOC samples. However, for this study, we are interested in (1) lineage-associated markers, *i.e.,* exo-protein biomarkers present in FT epithelium that are preserved in HGSOC, and (2) exo-protein biomarkers that would be suitable for both immunocapture and on chip detection using our microfluidic ExoProfile chip. Therefore, we focused on prioritizing exo-proteins within the 985 FT/HGSOC core proteome which are more likely to be displayed on the surface of EVs, thus making them suitable biomarker for screening and early detection. For this analysis, we intersected the 985 FT/HGSOC core proteome with known or predicted transmembrane proteins curated in the protein sequence database of UniProtKB (Swiss-Prot, July 2021—Supplementary File 4), which resulted in a truncated list of 75 exo-proteins (Fig. 2g & Supplementary Table 1). We then subtracted common EV proteins by comparing to the list of top 100 EV-associated proteins found in ExoCarta and Vesiclepedia databases. This narrowed the list to 66 transmembrane exo-proteins not commonly observed in EVs (Fig. 2h). We calculated the fold differences for these 66 proteins in the FT- and HGSOC-derived EVs and selected those that showed a log_2_ fold-change ≥ − 0.58 to identify proteins present in the FT samples and which increase in expression as the disease progresses to HGSOC (Supplementary Table 1). This approach resulted in a ranked list of 47 exo-proteins. Several of these proteins are integrins which have been implicated in cancer cell proliferation, migration and invasion^43^. Integrins have also been shown to be essential for EV homing and act as seeds that condition the favorable formation of tumor niches^24,44^. In addition, two of these proteins, IGHM and ADAM10, were found to be common EV proteins reported in literature^34,35,45^, which were not previously in listed in the ExoCarta and Vesiclepedia databases so they were manually removed from further analysis.

### Orthogonal analysis of LC–MS/MS detected transmembrane proteins by capillary western blot

**Figure 3:**
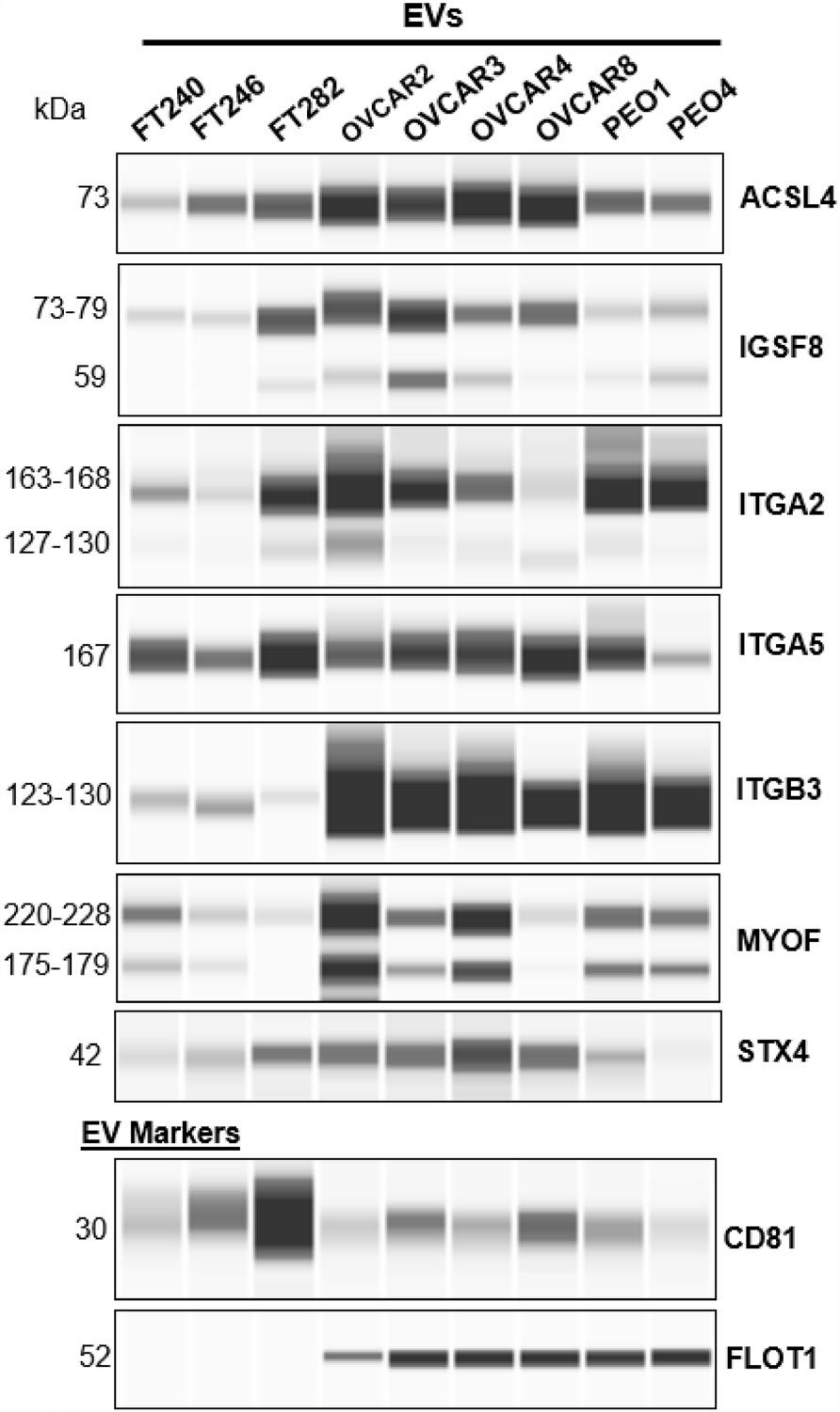
Detection of predicted transmembrane proteins in FT and HGSOC cell line EVs using capillary western blotting. One antibody for each of the 45 candidate transmembrane proteins was evaluated by capillary western blotting. The 7 exo-proteins that were confirmed to be present in EVs from 6 HGSOC and 3 FT cell lines are shown. Excluded from the figure are 23 exo-proteins with non-specific bands or no detectable bands at the expected molecular weight, and 15 exo-proteins with bands at the expected molecular weight but did not meet the criteria for presence in the EVs of all tested cell lines. In addition, CD81 and FLOT1 were evaluated as these are common EV markers. Full length unprocessed blots can be found in Supplementary Fig. 6.

We performed capillary western blotting (Simple Western—Wes platform) of the 45 transmembrane proteins (Supplementary Table 1) using one antibody per protein to confirm their presence within cell line derived EVs to support the LC–MS/MS data. Among the proteins analyzed using Wes, ACSL4 (FACL4), IGSF8 (CD316), ITGA2 (CD49b), ITGA5 (CD49e), ITGB3 (CD61), MYOF and STX4 were consistently detected in all the tested FT and HGSOC cell line EVs (Fig. 3 & Supplementary Fig. 6). These identified exo-protein biomarkers were prioritized for further analysis. However, for 23 candidate exo-protein biomarkers, we observed non-specific bands that did not correspond to the expected molecular weight, while others did not show any detectable bands. Although unable to validate due to technical limitations, these proteins might still be informative exo-protein biomarkers for future consideration. Additionally, although 15 exo-proteins showed bands at the expected molecular weight, these failed to meet our strict criteria: for lineage specific biomarkers, *i.e.,* must be detected in EVs derived from both FT and HGSOC cell lines used in our studies (**data not shown**). Therefore, these were also excluded from further validation.

### Evaluation of candidate biomarker transmembrane proteins in clinical samples

**Figure 4:**
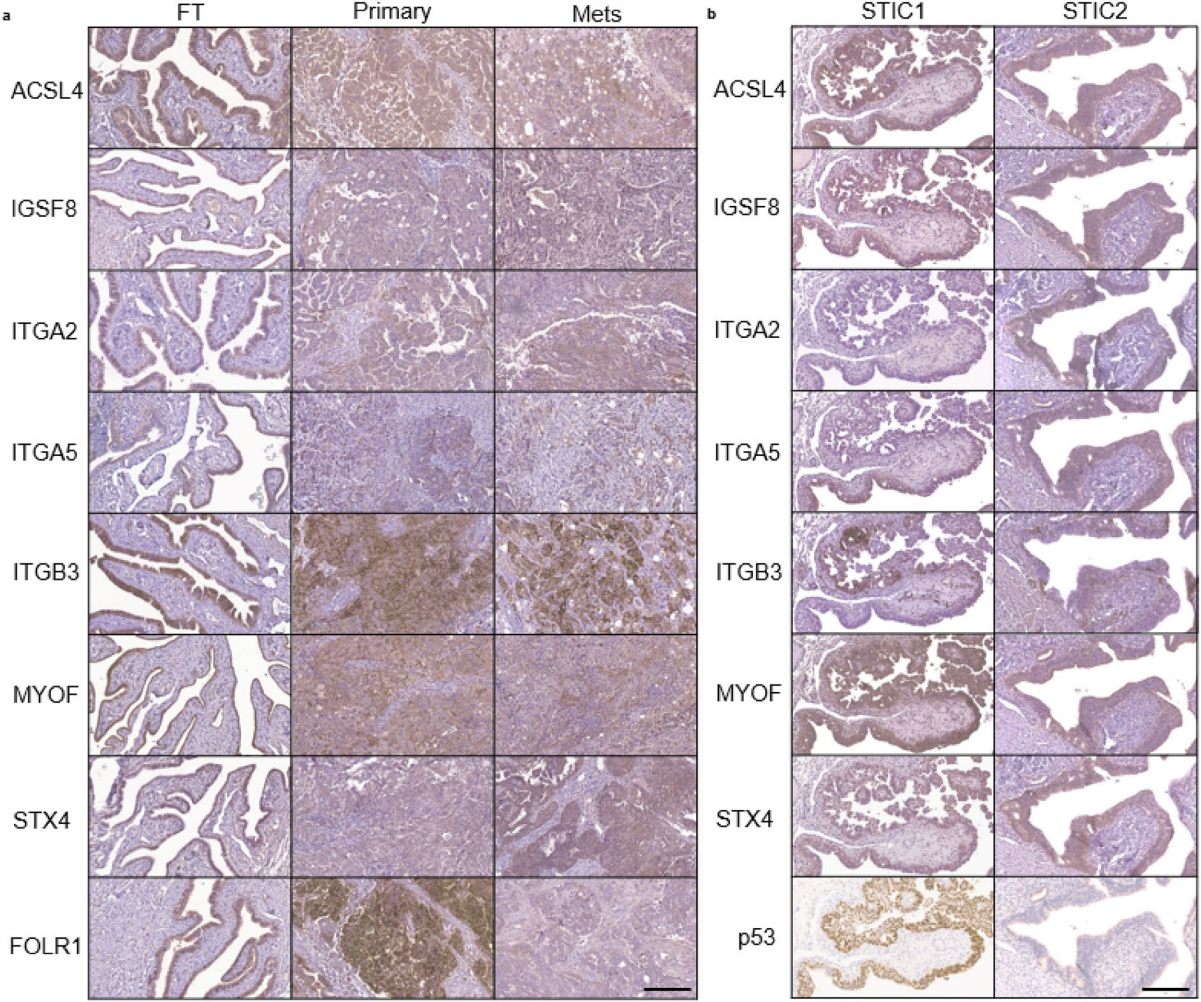
Immunohistochemistry staining of tissues from patients with HGSOC and FT tissue with STICs show expression of the candidate transmembrane proteins. (**a**) Representative IHC images from the tissue microarrays consisting of 100 patient samples containing benign FT, primary, and metastatic tumor tissue sections are shown for all markers except ITGA2; for ITGA2, tissue samples with higher IHC scores were selected for this figure. Supplementary Fig. 7 shows the IHC scores of all tissue samples used in the study. FOLR1 was included as a positive control for IHC staining. (**b**) p53-overexpressed STIC and p53-null STIC tissue sections from RRSO. p53 staining was done using an automated Dako Autostainer Link; a manual staining protocol was performed for the other markers. The scale bars represent 200 µm. Macro-tissue arrays of tissue sections representative of HGSOC, kidney, liver, placenta, spleen and tonsil were used as negative and positive controls. These macro-tissue arrays were also used in optimizing the antibody concentrations.

Since these seven transmembrane exo-proteins (ACSL4, IGSF8, ITGA2, ITGA5, ITGB3, MYOF and STX4) are present in FT and HGSOC cell lines, we next measured the expression of these proteins via immunohistochemistry (IHC) to confirm the tissues of origin. We created a tissue microarray (TMA) using samples from 100 patients with most of the samples having matching primary tumor, metastatic tumor, and a healthy region of fallopian tube tissue (Supplementary File 1). Following staining, a pathologist reviewed and scored each core (Fig. 4a & Supplementary Fig. 7). We found that all transmembrane proteins were expressed to varying degrees in healthy FT tissue, and in both primary and metastatic tumors. We included FOLR1 as a positive control since it has been shown to be highly expressed in ovarian tumors compared to healthy tissue but is decreased in platinum-resistant ovarian tumors compared to drug-sensitive tumors^46,47^. ITGA2 (CD49b) showed lower expression in all tissues compared to all the other proteins. Furthermore, we stained FT tissues with STIC lesions, based on being the precursors for most HGSOCs, obtained from risk-reducing salpingo-oopherectomy (RRSO) specimens. We found that in both p53-overexpressed and p53-null STICs, most of the candidate biomarker proteins were expressed in these regions. Interestingly, ITGA2 was found to be only expressed in the p53-null STIC, while ITGB3 (CD61) and ITGA5 (CD49e) demonstrated patches of staining within STIC lesions (Fig. 4b). The IHC protein expression results continued to support our hypothesis that these transmembrane proteins are present in the fallopian tube tissue and maintained during disease progression.

**Figure 5:**
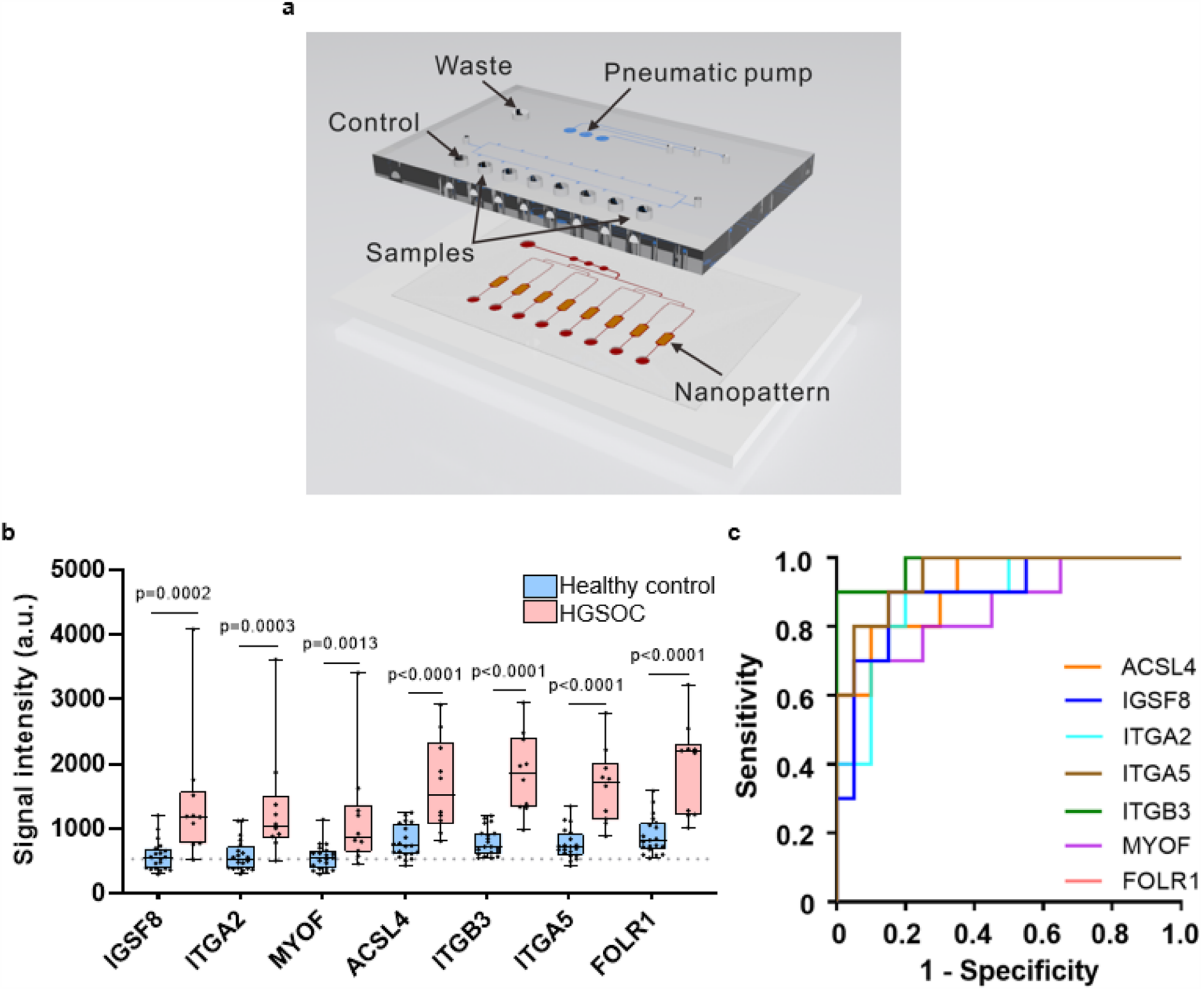
Evaluation of transmembrane exo-proteins in plasma samples using ExoProfile chips. (**a**) Image of an ExoProfile chip consisting of 8 nanopatterned parallel channels for EV capture. (**b**) Quantification of transmembrane exo-protein biomarkers on captured CD81 + EVs for HGSOC (n = 10) and healthy control (n = 20) (dotted line signifies background fluorescence from a negative control channel labeled as BKG). FOLR1 was included as a previous positive control for HGSOC^32^. *p*-values were calculated using Mann–Whitney U test. (**c**) Area under the curve plot of receiver operating characteristic analyses for all the six markers and FOLR1 are shown.

After confirming that these transmembrane proteins are present in tissues via IHC, we then tested these exo-proteins in patient plasma samples using a modified ExoProfile (Fig. 5a) microfluidic chip capable of EV immunocapture and fluorescence detection^32^. We used this functionalized nano-engineered microfluidic platform to evaluate the diagnostic power of the FT/HGSOC exo-protein biomarkers. Based on our previous publications, exo-FOLR1 was able to differentiate ovarian patients (late and early stage) from benign and healthy controls with high specificity and sensitivity^32,48^. Therefore, we used FOLR1 as the positive control to assess the specificity and sensitivity of the current lineage specific biomarkers being evaluated. Using the ExoProfile chip, specifically developed to simultaneously measure multiple EV-associated analytes, we captured CD81 + EVs from plasma (n = 10 cases and n = 20 age/race matched controls—Supplementary File 1) and quantified the relative levels of ACSL4, IGSF8, ITGA2, ITGA5, ITGB3, MYOF, and FOLR1 (Fig. 5b**,** unfortunately we were not able to test STX4 since an antibody compatible with the ExoProfile chip was not available). The plasma samples were obtained from HGSOC patients with both early (FIGO Stage I–II; n = 5) and advanced stage (FIGO Stage III–IV, n = 5) disease. Notably, all seven FT/HGSOC exo-protein biomarkers were detected at significantly higher levels in the HGSOC plasma (regardless of stages) relative to healthy plasma having *p*-values ≤ 0.001. We performed ROC analysis for each marker and found that four markers (ACSL4, ITGB3, ITGA5 and FOLR1) had an area under the ROC curve (AUC) that is higher for early stage compared to healthy plasma versus late stage compared to healthy plasma (Supplementary Table 2). Since this sample set of diseased patients is relatively small, we proceeded with calculating all the ovarian cancer patients (n = 10) together instead of in separate groups (early or late stage) against the healthy controls (n = 20). We found that these markers had AUCs ranging from 0.85 to 0.98 (Fig. 5c & Table 1), which indicates a significant separation between diseased and healthy controls. In fact, exo-ITGA5 and exo-ITGB3 (AUC of 0.95 and 0.98, respectively) performed better than exo-FOLR1 (AUC of 0.925) which was a robust ovarian cancer biomarker based on our previous work^32^. We also performed various marker combinations of two or more markers and found that the linear combination of IGSF8 and ITGA5 based on logistic regression analysis yielded an AUC of 0.990 with a sensitivity of 0.80 at 99.8% specificity (Table 1 & data not shown). Based on multivariable logistic regression analysis, we scrutinized for all possible logistic regression models that can accommodate any subset of the 7 markers and selected the best based on the Akaike Information Criterion (AIC). The equation we derived for the best marker combination is as follows: Linear combination of IGSF8 & ITGA5 = 11.299 × log(IGSF8) + 14.935 × log(ITGA5). Furthermore, we calculated the positive predictive value (PPV) for each individual marker, as well as for the linear combination of IGSF8 and ITGA5 (Table 1). The PPV is a measure that can assess the predictive ability of the marker, in particular it is the probability of having the disease given a positive test result^49^. For our sample set, we achieved a PPV ranging from 5.7 to 13.8%, using a prevalence of 1/2500 and a cutoff that forces a specificity of 0.998. Notably, the linear combination of IGSF8 and ITGA5 showed exceptional performance, with a remarkably high PPV of 13.8% along with increased sensitivity at 0.80 and a forced specificity of 99.8%. Our study surpassed the minimum performance of at least 10% PPV for ovarian cancer screening studies^50^. We next focused on how specific these biomarkers are to ovarian cancers.

**Table 1:** Statistical analysis for each exo-protein biomarker including the most sensitive linear marker combination of IGSF8 and ITGA5 on the ExoProfile chip.

Exo-protein	AUC	Standard error	CI_lower	CI_upper	*p*-value-1t	Sensitivity at 99.8% specificity	PPV
IGSF8	0.895	0.064	0.770	1.000	2.77 × 10^–4^	0.300	0.057
ITGA2	0.885	0.064	0.759	1.000	3.82 × 10^–4^	0.400	0.074
MYOF	0.850	0.080	0.692	1.000	1.12 × 10^–3^	0.400	0.074
ACSL4	0.915	0.052	0.813	1.000	1.42 × 10^–4^	0.600	0.107
ITGB3	0.980	0.022	0.938	1.000	1.33 × 10^–4^	0.900	0.153
ITGA5	0.950	0.037	0.878	1.000	4.12 × 10^–5^	0.600	0.107
FOLR1	0.925	0.047	0.832	1.000	1.01 × 10^–4^	0.600	0.107
Linear combination of IGSF8 & ITGA5	0.990	0.020	0.964	1.000	1.33 × 10^–5^	0.800	0.138

**Figure 6:**
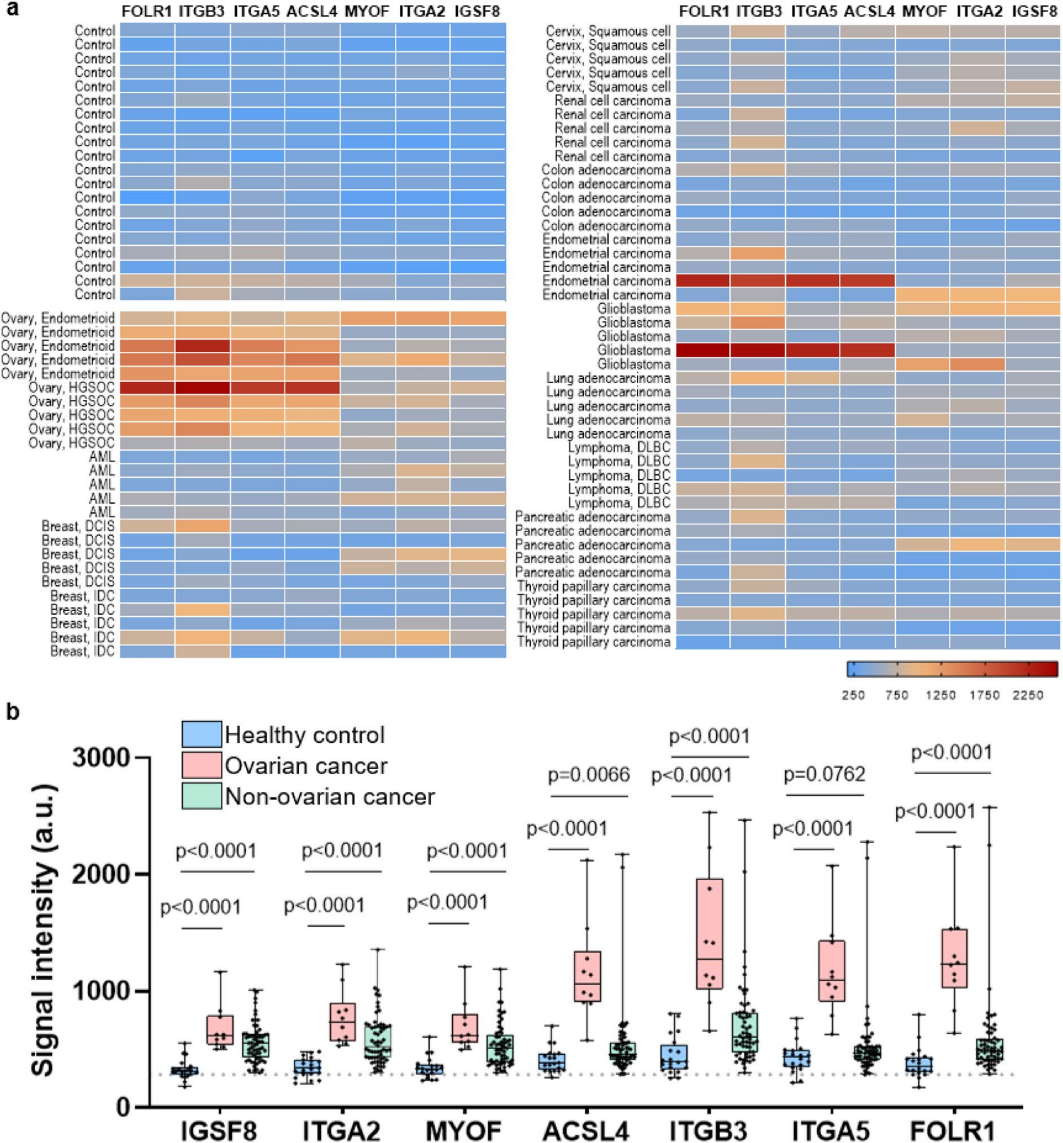
Specificity testing of transmembrane exo-proteins in plasma samples from different cancer types using the ExoProfile chip. (**a**) Heatmap showing the relative intensity distribution of each exo-protein marker across 90 plasma samples comprising of 12 non-ovarian cancers commonly diagnosed in women (n = 60; 5 per each cancer type), ovarian cancer (n = 10) and healthy controls (n = 20). (**b**) Quantification and comparison of transmembrane exo-protein biomarkers on captured CD81 + EVs in non-ovarian cancers (n = 60), ovarian cancers (n = 10), and healthy control samples (n = 20). The dotted line represents background fluorescence from a negative control channel. *p*-values were calculated using Mann–Whitney U test. These samples were subjected to two separate tests on different chips, and the reported values represent the average of two experiments. AML: acute myeloid leukemia, DCIS: ductal carcinoma in situ, IDC: invasive ductal carcinoma, DLBC: diffuse large B cell lymphoma.

To address this question, we conducted further testing on 90 additional plasma samples, which included 12 cancers that are commonly diagnosed in women (5 samples per type), 10 ovarian cancers (endometrioid and HGSOC subtypes) and 20 female healthy controls. We observed in this independent set of samples that FOLR1, ITGB3, ITGA5 and ACSL4 have higher expression for majority of the ovarian cancer cases compared to the non-ovarian cases (Fig. 6a). Interestingly, the patient sample which exhibited a lower expression of FOLR1, which serves as the positive control for HGSOC, also showed reduced expression levels for ITGB3, ITGA5 and ACSL4. Furthermore, when quantitating the CA-125 levels in aliquots of the same plasma samples used on the ExoProfile chip, this outlier had the lowest levels among the HGSOC cases (8.9 U/mL; data not shown), suggesting a lower tumor burden when the blood was collected. We performed group comparisons of healthy controls versus ovarian cancer, and healthy controls versus non-ovarian cancer using Mann–Whitney U test. The *p*-values for each individual marker indicated statistically significant differences when comparing healthy versus ovarian cancer samples. However, in the non-ovarian versus healthy controls, ACSL4 (*p* = 0.0066) and ITGA5 (*p* = 0.0762) did not achieve statistical significance at a nominal level of 0.05 (Fig. 6b). Additionally, to validate our findings, we applied the fixed linear combination marker derived from the smaller sample set of 10 ovarian cancer cases and 20 healthy controls in the previous experiment to this specificity dataset. Using the linear combination of IGSF8 and ITGA5 on the ovarian cancer (n = 10) and healthy control samples (n = 20) on this specificity data set, we achieved a PPV of 15.3% with a sensitivity of 0.90 at specificity of 99.8%, confirming the reliability of our original marker set (Supplementary Table 3). Our study demonstrates promising results with the linear combination of IGSF8 and ITGA5 showing exceptional performance in terms of PPV, sensitivity at a forced level of 99.8% specificity. These results confirm that the lineage associated exo-protein biomarkers detected from proteomic profiling of FT/HGSOC tissue-derived EVs can be incorporated onto the ExoProfile chip to develop a clinically relevant liquid biopsy test focused on the early detection of this disease, and potentially while confined to the fallopian tubes.

## Discussion

Developing non-invasive and highly specific blood-based test for pre-symptomatic screening and early detection of ovarian cancer is crucial since no clinical trials have been successful in reducing the mortality rates of women with EOC^17^, and the currently available blood-based biomarkers have low specificity. Ultimately, our goal is to develop a highly specific test that can detect HGSOC, the deadliest form of ovarian cancer, at its earlier stages, especially in women who are at increased risk of developing ovarian cancer (*e.g., BRCA1* and *BRCA2* mutation carriers). When translated into the clinic, these types of liquid-based assays can help identify at risk women in need of risk reducing salpingectomy (surgical removal of the fallopian tubes) without oophorectomy (removal of the ovaries). Not only would this approach reduce the their change of developing a deadly cancer, but could do so while retaining hormone production^7^. As reported, we have for the first time identified candidate exo-biomarkers focused on lineage specific proteins shared between fallopian tube epithelium and HGSOC. We demonstrated that these new ovarian cancer biomarkers could discriminate ovarian cancer cases from healthy controls. In fact, when we performed a separate analysis of the early stage compared to late stage versus healthy, four markers (ACSL4, ITGB3, ITGA5 and FOLR1) demonstrated higher AUC values (Supplementary Table 2).

Several studies on various diseases have performed proteomic profiling on EVs since these contain valuable information which can be translated into noninvasive diagnostic platforms^27^. In HGSOC, initial proteomic studies on EVs were performed on a few cell lines^51^. A study by Selvendiran and colleagues performed proteomic profiling on CD63 + and EpCAM + EVs enriched using a microfluidic device from HGSOC, FTSEC and ovarian surface epithelial (OSE) cell lines but these studies were dependent on a single cell line per each type (OVCAR8, FT33 and OSE). From this study, IL-6, HGF and STAT3 were identified to be highly expressed in EVs from ovarian cancer patient serum^52^. EVs circulating in the blood can originate from any cell type either healthy or diseased. Thus, there is a growing need to define which subpopulations are significant to study. We hypothesized that evaluating protein expression on EVs found in FT tissue and shared with HGSOC cells has the capability to develop a clinically relevant assay to detect HGSOC at its earliest stages, *i.e.,* while still confined to the fallopian tube.

One study has published an in silico approach to identify membrane proteins by combining several databases namely, the Cancer Genome Atlas Research Network (TCGA), Genotype-Tissue Expression Project (GTEx) and the Human Protein Atlas^53^. Their study focused on predicted transmembrane proteins that have higher expression in HGSOC tissues compared to healthy tissues, while our study was more focused on shared exo-proteins between FT and HGSOC. When we compared their list of 1451 transmembrane proteins identified via in silico approach^53^ with our list of the 45 transmembrane exo-proteins (Supplementary Table 1), we found that more than 50% of the EV transmembrane proteins (n = 27) were in common between the two data sets. More importantly, we identified 18 unique exo-proteins that were solely found in our EV data not previously discovered (Supplementary Table 1). Previously, this comparison was not possible because our study is the first to generate a comprehensive analysis of FT/HGSOC EV proteome from tissues and cell lines. ACSL4 and ITGB3 were part of these 18 unique exo-proteins, both of which were able to discriminate healthy from diseased patients using the ExoProfile assay.

Based on the exo-protein biomarkers identified with the workflow we employed, we were able to narrow down the markers to seven proteins namely, ACSL4, IGSF8, ITGA2, ITGA5, ITGB3, MYOF and STX4. Previous studies on protein levels of these markers have shown that ACSL4, ITGA5 and STX4 are highly upregulated in ovarian cancer tissues^54–56^, while ITGA2 was upregulated in omental tumors^57^. High protein expression levels of ACSL4, ITGA2 and ITGA5 have been implicated in poor prognosis^54,55,58^; however, the low expression of ITGB3 in HGSOC has been associated with favorable prognosis^59^. Moreover, ITGA2 has been shown to impart paclitaxel resistance^58^, while ITGA5 from ascitic tumor cells is essential for cancer associated fibroblasts to initiate peritoneal metastasis^60^. Interestingly, neither IGSF8 nor MYOF have been previously implicated in HGSOC or fallopian tube biology but MYOF in EVs from breast and pancreatic cancer cell lines has been shown to contribute to cancer progression via metastasis^61^. Although most of these markers appear to have been studied in ovarian cancer biology, our study results are the first to report their presence in circulating EVs from clinical ovarian cancer blood samples and show their utility in detecting ovarian cancer at early stages and with high specificity.

A caveat of this study is that most of the ovarian cancer patients’ tissue samples obtained as short-term explants had been collected after neoadjuvant chemotherapy since this is the standard of care before performing a debulking surgery. The ideal situation would be to obtain tumor tissues from untreated patients, which could yield a slightly modified EV-associated proteomic profile. We are aware of the possibility that systemic host responses from immune cell activation could influence EV cargo, as well as tumor cellularity since these tissues would have varying amounts of tumor present in the tissues that we processed. This is specifically why we used well characterized FT and HGSOC cell lines to confirm the epithelial origin of the biomarkers. Also, these studies did not assess post-translational modifications such as glycosylation which typically occur in cancers. While CD81 (as well as CD9 and CD63) is not universally present in all EVs, our previous work has shown that preselecting a subpopulation of plasma EVs using CD81 can reproducibly detect ovarian cancer-associated biomarkers using these nano-engineered microfluidic chips^30,32^. Moving forward, collaborative efforts will be needed to expand the clinical samples, focusing on blood samples from women prior to risk reducing surgery who were diagnosed upon pathological examination with STIC lesions. Despite these limitations, our study is the first to establish the protein content of tissue derived EVs from healthy fallopian tube, primary HGSOC and omental metastatic cancer tissues. Our focus was to identify lineage associated exo-proteins predicted to be transmembrane for immunocapture and detection which can advance clinical diagnostic applications. We have identified transmembrane proteins associated uniquely with FT and HGSOC EVs. When these protein markers are used with our functionalized ExoProfile chip, we were able to distinguish the HGSOC patients (both early and late stage equally) from matched healthy individuals with high AUC values. When we combined IGSF8 and ITGA5, we achieved a sensitivity of 0.80 at 99.8% specificity with a PPV of 13.8%. This performance was further validated when we utilized the same combination as a training set and applied it to a separate specificity data set. In this validation, we observed an even higher sensitivity of 0.90 at a specificity of 99.8% with a PPV of 15.3% and the ability to specifically detect ovarian disease among other common cancers diagnosed in women. These findings were consistently replicated indicating the robustness and reliability of our original marker set. These results exceed the performance of clinically adopted serum multimarker panels, *i.e.*, CA-125 (gold standard) and HE4^62,63^, which emphasizes the clinical significance of the biomarker combinations in the context of HGSOC screening.

Although this study was performed on a small number of early stage HGSOC cases, and a limited number of non-ovarian cancer cases (5 per cancer type), future studies are clearly warranted to validate the sensitivity of these exo-biomarkers in clinical samples from asymptomatic-high risk women who subsequently are diagnosed with HGSOC. In summary, this study underscores the potential clinical utility of our newly discovered protein biomarkers, particularly IGSF8 and ITGA5, for HGSOC screening.

## Material and methods

### Human samples

De-identified plasma samples from healthy and untreated HGSOC patients with early (FIGO Stage I–II) or advanced stage (FIGO Stage III-IV) disease were obtained from the University of Kansas Medical Center Biospecimen Repository Core Facility (KUMC BRCF). Biospecimens are obtained using a protocol approved by the University of Kansas Medical Center’s Internal Review Board. The KUMC BRCF was established to obtain tumor and blood specimens from informed consented patients undergoing treatment at our institution as well as healthy volunteers as part of a protocol approved by our internal Human Subjects Committee (HSC #5929) and following U.S. Common Rule. The studies have been approved by the appropriate institutional research ethics committee and have been performed in accordance with the ethical standards as laid down in the 1964 Declaration of Helsinki and its later amendments or comparable ethical standards.

Primary HGSOCs tumors or metastatic tissue from the omentum were obtained from women with Stage II–IV HGSOCs who were undergoing tumor debulking surgery. Healthy FT tissues were obtained from patients undergoing salpingo-oophorectomy for various medical conditions, including hysterectomies for non-cancerous conditions. Archival formalin-fixed paraffin-embedded (FFPE) FT tissue samples with STIC lesions were obtained from women undergoing RRSO after pathological review. Informed consent was obtained from all participants included in the study. The de-identified tissues obtained were minced into small pieces and placed in 6-well plates containing 3 mL of cell media. Cell media without supplements and without serum was added and placed in a humidified incubator at 37 °C with 5% CO_2_ for 24 h. The conditioned media was collected and EVs were enriched from the media using differential ultracentrifugation as described below.

### Cell culture

FT cell lines, FT240, FT246 and FT282^64^ were a kind gift from Dr. Ronny Drapkin (University of Pennsylvania). All cell lines were validated by short tandem repeat fingerprinting. FT cell lines were cultured in a 50/50 mixture of DMEM/F-12 without L-glutamine (Corning) supplemented with 2% (v/v) Ultroser G (Pall Biosciences) and 1% (v/v) penicillin–streptomycin at 37 °C with 5% CO_2_. UltroserG was ultracentrifuged for a minimum of 18 h at 100,000×g followed by filtration through a 0.2 µm filter. Ovarian cancer cell lines (OVCAR2, OVCAR3, OVCAR4, OVCAR8, PEO1 and PEO4) were cultured in RPMI1640 media (Hyclone, Cytiva Life Sciences) supplemented with 10% (v/v) EV-depleted FBS (spun at 100,000×g at 4 °C for at least 18 h and filtered using a 0.2 µm filter), 2.5% mg/mL insulin and 100 units/mL penicillin–streptomycin at 37 °C with 5% CO_2_. Conditioned media was collected when cells were at least 60% confluent. Conditioned media from each cell line was collected from either two separate passages (for FT cells) or three separate passages (for HGSOC cells). Each collection was processed and maintained as an independent biological replicate even throughout the LC–MS/MS procedure and analysis.

### Enrichment of EVs from conditioned media by differential ultracentrifugation

Conditioned media from cell cultures and tissue explants was collected and centrifuged at 300×g for 10 min to remove cell debris. The supernatant fraction was then centrifuged at 2000×g for 20 min to remove apoptotic bodies. This was followed with supernatant centrifugation at 10,000×g to remove large microvesicles for 1 h followed by 100,000×g spin to collect EVs. The EV pellets were washed and resuspended in PBS and spun for 1 h at 100,000×g. The EV pellets were resuspended in PBS and stored at − 80 °C.

### Transmission electron microscopy

Glow-discharged carbon-coated copper grids were floated on the surface of a drop of 30 µL of EVs for 20 min. The grids were then rinsed with water followed by negative staining with 1% uranyl acetate for 5 s. Once the grids were dry, TEM images were taken using a JEM-1400 Transmission Electron Microscope (JEOL USA, Inc.) equipped with a Lab6 gun.

### Nanoparticle tracking analysis (NTA)

The concentration and size of the enriched EVs were analyzed using the NanoSight LM10 instrument (Malvern Panalytical Ltd). NTA was performed using a monochromatic 404 nm laser on EVs diluted in 0.2 µm filtered PBS. Three recordings of 60 s videos were taken per sample at camera level 13 using the NTA software version 2.3. Data was compiled using a custom MATLAB code.

### Single particle interferometric reflectance imaging sensing and fluorescence

Enriched EV samples (17.5 µL each) were mixed with an equal volume of Solution A. The sample was then place on an ExoView chip and incubated overnight. 1 mL of Solution A was added, and this was shaken at 500 rpm for 3 min at room temperature. The chip was then washed three-times with incubation solution before adding the blocking solution containing tetraspanin antibodies (CD9, CD63 and CD81). The chips were incubated for 1 h at room temperature. Incubation solution was added and shaking at 500 rpm for 3 min at room temperature was repeated. The ExoView chip was washed 3 times before adding rinse solution and was scanned using the ExoView R100 instrument (NanoView Biosciences). nScan 2.8.10 software was used for data acquisition and NanoViewer software was used for data analysis (both from NanoView Biosciences). The threshold used for cut-off was 500 arbitrary units of fluorescence for the red, green, and blue channel in all experiments.

### LC-MS/MS

Enriched EV samples were submitted to the Proteomics and Mass Spectrometry Facility at the Donald Danforth Plant Science Center (St. Louis, MO) for LC–MS/MS analysis. Twenty micrograms of EVs per sample were denatured using 8 M urea, reduced with 10 mM TCEP, and alkylated with 25 mM iodoacetamide followed by digestion with trypsin/Lys-C mix at 37 °C overnight. The digested sample was acidified with 1% TFA then cleaned up using a Pierce C18 tip (Thermo-Fisher Scientific). The extracted peptides were dried down and each sample was resuspended in 30 μL 1% acetonitrile/1% formic acid. Approximately 1 µg of each sample was injected for LC–MS/MS analysis.

LC–MS/MS was carried out on an Orbitrap Fusion Lumos (Thermo Fisher Scientific) mass spectrometer coupled with a U3000 RSLCnano HPLC (Thermo Fisher Scientific). The peptide separation was carried out on a C18 column (Acclaim PepMap RSLC, 50 cm × 75 μm nanoViper™, C18, 2 μm, 100 Å, Thermo Fisher Scientific) at a flow rate of 0.3 μL/min and the following gradient: Time = 0–4 min, 2% B isocratic; 4–8 min, 2–10% B; 8–83 min, 10–25% B; 83–97 min, 25–50% B; 97–105 min, 50–98%. Mobile phase A consisted of 0.1% formic acid and mobile phase B consisted of 0.1% formic acid in acetonitrile. The instrument was operated in the data-dependent acquisition mode in which each MS1 scan was followed by higher-energy collisional dissociation (HCD) of as many precursor ions in a 2-s cycle (Top Speed method). The mass range for the MS1 done using the FTMS was 365–1800 m/z with resolving power set to 60,000 @ 400 m/z and the automatic gain control (AGC) target set to 1,000,000 ions with a maximum fill time of 100 ms. The selected precursors were fragmented in the ion trap using an isolation window of 1.5 m/z, an AGC target value of 10,000 ions, a maximum fill time of 100 ms, a normalized collision energy of 35 and activation time of 30 ms. Dynamic exclusion was performed with a repeat count of 1, exclusion duration of 30 s, and a minimum MS ion count for triggering MS/MS set to 5000 counts.

### Identification and label free quantification of proteins

Sequence mapping and label-free quantification were done using Proteome Discoverer (PD) version 2.4 (Thermo Fisher Scientific). Database searches with Sequest search engine were launched in PD and queried against Human reference proteome (Uniprot.org, April 2021). The digestion enzyme was set as trypsin. The MS/MS spectra were searched with a precursor mass tolerance of 10 ppm and a fragment ion mass tolerance of 0.6 Da. Carbamidomethylation of cysteine was set as fixed modification. Oxidation of methionine and acetylation of N-terminal of protein were specified as variable modifications. Matched peptides were filtered using a Percolator-based 1% false discovery rate (FDR). Protein quantification was achieved by using total intensities of all precursors.

### Simple Western assay (Wes)

The concentration of EV proteins was established using the Bradford assay (Bio-Rad) according to manufacturer’s instructions. Simple Western assay (Wes, ProteinSimple) was used for the detection of EV markers (CD81 and Flotillin-1) and proteins that were selected for further evaluation, namely ACSL4, IGSF8, ITGA2, ITGA5, ITGB3, MYOF and STX4 (for additional details please see Supplementary Table 4). EVs at a concentration of 0.4 µg/µL were used in these assays. The 12–230 kDa or 66–440 kDa Wes separation module and the secondary anti-mouse, anti-rabbit, and anti-goat detection modules were used following manufacturer’s instructions. Chemiluminescent detection profile was set at High Dynamic Range 4.0 and contrast was manually adjusted for each sample. Data were analyzed using the Compass software version 6.0.0. (ProteinSimple).

### Immunohistochemistry staining of TMA and STICs

Unstained tissue slide sections of five TMA blocks containing representative tissue cores of benign fallopian tube, primary and metastatic ovarian tumor tissues (n = 100) were provided by the KUMC BRCF. Tissue microarrays were previously constructed by the BRCF staff using archival FFPE tissue blocks and were provided as a kind gift by Dr. Dineo Khabele (Washington University, St. Louis). Tissue sections were placed in xylene and rehydrated in ethanol baths of decreasing concentration. Antigen retrieval was performed by heating with citrate buffer in a pressure cooker for 15 min. Once at room temperature, endogenous peroxidase activity was blocked using BLOXALL blocking solution (Vector Laboratories) for 20 min, slides were washed followed by another blocking step using 2.5% normal horse serum for 30 min. Primary antibody incubation using ACSL4, IGSF8, ITGA2, ITGA5, ITGB3, MYOF, STX4 and FOLR1 was performed overnight (for additional details please see Supplementary Table 4). Anti-mouse, anti-rabbit, or anti-goat secondary ImmPRESS horse IgG polymer reagent (Vector Laboratories) was added and incubated for 30 min. The ImmPACT DAB EqV reagent was then incubated for 1–5 min. A light hematoxylin counterstain was performed followed by dehydration, clearing, and mounting using permanent mounting medium (Vector Laboratories). These stained slides were then visualized under a bright field microscope and scored by a board-certified pathologist using the following formula: H-score = (0 × area of cells with absent staining) + (1 × area of “1+” cells%) + (2 × area “2+” cells%) + (3 × area “3+” cells%)^65^.

FT tissue sections containing STIC lesions were stained for p53 using an automated protocol optimized for the Dako Autostainer Link (Agilent) or for the candidate protein biomarkers using the manual IHC staining protocol described above.

### EV immunoassay using the ExoProfile chip

The ExoProfile chip was functionalized for antibody conjugation and anti-CD81 antibody was flowed through the chip to coat the surface similar to previous methods^32^. De-identified plasma samples, first processed to remove platelets (2500×g for 15 min), were centrifuged at 10,000×g for 30 min at 4 °C to remove large microvesicles. 10 µL of plasma was diluted to 100 µL and used to detect seven exo-protein biomarkers (ACSL4, IGSF8, ITGA2, ITGA5, ITGB3, FOLR1 and MYOF) simultaneously. One channel was designated as the negative control (PBS) to measure any background fluorescence. 10 µL of 1:10 diluted plasma was flowed through the chip with a constant flow rate of 5 µL/h. Assays were performed on the ExoProfile chip similar to those described previously^32^. Fluorescence images were captured using a Nikon Ti2 inverted fluorescence microscope equipped with a LED excitation light source (Lumencor). Fluorescence intensity was quantified by processing and analyzing the images using ImageJ.

### Bioinformatics analysis

Qualitative analysis (GO identification and network analysis) of the FT/HGSOC core proteome was analyzed using DAVID version 6.8. The protein–protein interaction network was obtained using STRING version 11.5. *p*-values were calculated using EdgeR analysis^66^. The log_2_ fold change values and the adjusted *p*-values were used to define significant differential expression proteins. Qprot^67^ v1.3.5 was used to calculate Z-statistics, log_2_(fold change) and FDR values. Qprot analysis was performed with a burn-in value of 2000 and 10,000 iterations.

To identify monotone and non-monotone markers, we used the length of the ROC curve^42^. The length of the ROC curve for each protein was estimated non-parametrically using a Gaussian kernel density estimator for the scores of each group (FT and HGSOC), so that a smooth ROC curve estimate is available. Monotone markers are proteins which have an ROC curve length of 2 and exhibit AUC > 0.8 and AUC < 0.2, while non-monotone markers are proteins that have an ROC curve length of > 1.6 and exhibit AUC ≥ 0.35 but ≤ 0.65.

### Statistical analysis

For NTA data and IHC expression score analysis, GraphPad Prism version 8 was used for performing Mann–Whitney U tests in calculating *p*-values to compare between the samples. Data were expressed as means ± s.e.m. (standard error of mean). For the ExoProfile chip, one-way ANOVA was performed on the fluorescent intensities measured per each sample per biomarker. ROC/AUC for single marker analysis was performed using GraphPad Prism version 8. For combination marker analysis, we explored all possible combinations of markers which involved scrutinizing all possible models with 2 markers, with 3 markers etc., along with a model that included all markers. The AIC was used to identify the best marker combination^68^. The linear term of this logistic regression model is then extracted to be utilized as the combined marker score. We then maximized the Youden index^69^ to derive the sensitivity and specificity at the optimized cutoff. The positive predictive values were calculated by using prevalence set at 1/2500 which is for postmenopausal women in the general population^70^.

### Supplementary Information

Supplementary Information 1.

Supplementary Information 2.

Supplementary Information 3.

Supplementary Information 4.

Supplementary Information 5.

Supplementary Information 6.

Supplementary Tables.

## Data Availability

Most of the data generated or analyzed in the study are available within the manuscript, supplementary materials, files and tables. The mass spectrometry proteomics data have been deposited on the Proteome Xchange Consortium via PRIDE^71^ with the dataset identifier PXD036726. The rest of the data are available from the corresponding author upon reasonable request.
