# Supplementary material for: Lineage specific extracellular vesicle-associated protein biomarkers for the early detection of high grade serous ovarian cancer
Source: Sci Rep. 2023 Oct 26;13:18341. doi: 10.1038/s41598-023-44050-5 (PMC10603107; doi:10.1038/s41598-023-44050-5)
Supplement: Supplementary file 3 — Supplementary Information 3. [file 41598_2023_44050_MOESM3_ESM.docx]

**Bioinformatic analysis of FT/HGSOC proteins identified via LC-MS/MS**

We performed GO analysis on the 985 core set of proteins using the Database for Annotation, Visualization and Integrated Discovery (DAVID)^1^ to identify enriched biological classes and functional-related gene groups and found that majority of these proteins were associated with EVs (65.8%) which was reassuring (**Supplementary File 5**). These proteins included common EV markers, *e.g.,* CD9, Alix, FLOT1, FLOT2, HSP70, HSC70, HSP90AA1, HSP90AB1 and SDCBP (**Fig. 2c**). Also, the majority of the proteins were reported to be identified to be cytosolic (51.3%) or membrane associated (35.6%) based on their predicted location in cells (**Supplementary Fig. 8a** **& Supplementary File 5**). In terms of biological process, several of these proteins are involved in translational initiation, cell-cell adhesion, SRP-dependent co-translational process in a cellular level (**Supplementary Fig. 8b & Supplementary File 5**). Most translational initiation and SRP-dependent co-translational proteins found in EVs include several ribosomal proteins (RPL: ribosomal protein large subunit and ribosomal protein small subunit RPS). RPL and RPS proteins are important in regulating cellular processes such as protein synthesis, cell growth and development^2,3^; however, their roles in EVs are not well defined. Several studies have shown the presence of RPL and RPS mRNA or proteins in EVs, namely in exosome-like vesicles from porcine follicular fluid^4^ and bovine oviductal EVs^5^. Ribosomal proteins have been suggested to be considered as potential biomarkers since dysregulation of these proteins can lead to various diseases such as cancers^6^. Proteins identified to play a role in cell-cell adhesion included EPCAM, ENO1 and SEPTIN2, all of which have previously been implicated in epithelial ovarian cancer development^7-9^. We also compared the FT/HGSOC core proteome to Vesiclepedia (a public EV proteome database) using FunRich version 3.1.3 and identified 52 unique proteins (**Supplementary Fig. 9**). These 52 proteins were primarily associated with nucleosome organization, tRNA aminoacylation or septins, a family of conserved GTPase (**Supplementary Fig. 10**). These analysis shows that the FT/HGSOC core proteome contain proteins that are commonly found in EVs, as well as proteins that would likely be characteristic of cells of HGSOC origin, *i.e.,* from the fallopian tube epithelium, which is of Müllerian derivation and subject to the same range of metaplastic change seen in other gynecologic sites (*e.g.,* cervix, endometrium).

We also performed ROC analysis on the 985 exo-proteins and identified a set of monotone and non-monotone markers that included non-transmembrane or cytosolic proteins to look for possible markers that may further be examined. Monotone markers are those that have an ROC length of 2 and exhibit AUC>0.8 and AUC<0.2, while non-monotone markers are defined as markers that might imply too high or too low levels for one group while their levels are in a range between two cutoffs for the other group (ROC length of >1.6 and 0.35 ≤ AUC ≤ 0.65). Using the cell line and tissue explant data, we found 27 monotone markers and 16 non-monotone markers in the FT/HGSOC core proteome (**Supplementary Table 5**). These are candidate markers that we plan to evaluate in clinical samples to identify late-stage disease from non-tumor samples.

We have also evaluated the 324 proteins that were uniquely identified in only the HGSOC samples but not present in FT (**Fig. 2F & Supplementary Table 6**). We assessed the biological functions of these protein by performing Metascape^10^ analysis (**Supplementary Fig. 11a-b)**. We found that these exo-proteins are enriched in proteins involved in RNA metabolism, cellular responses to stress, neutrophil degranulation and cytokine signaling in the immune system. Taken together, our comprehensive proteomic analysis including the 985 FT/HGSOC core proteome and the 324 proteins unique to HGSOC samples are a rich source of potential candidate biomarkers beyond the transmembrane EV proteins that we have extensively studied in the current manuscript.

**References**

1 Huang da, W., Sherman, B. T. & Lempicki, R. A. Systematic and integrative analysis of large gene lists using DAVID bioinformatics resources. *Nat Protoc* **4**, 44-57, doi:10.1038/nprot.2008.211 (2009).

2 Provost, E., Weier, C. A. & Leach, S. D. Multiple ribosomal proteins are expressed at high levels in developing zebrafish endoderm and are required for normal exocrine pancreas development. *Zebrafish* **10**, 161-169, doi:10.1089/zeb.2013.0884 (2013).

3 Zhou, X., Liao, W. J., Liao, J. M., Liao, P. & Lu, H. Ribosomal proteins: functions beyond the ribosome. *J Mol Cell Biol* **7**, 92-104, doi:10.1093/jmcb/mjv014 (2015).

4 Matsuno, Y. *et al.* Characterization of mRNA profiles of the exosome-like vesicles in porcine follicular fluid. *PLoS One* **14**, e0217760, doi:10.1371/journal.pone.0217760 (2019).

5 Alminana, C. *et al.* Deciphering the oviductal extracellular vesicles content across the estrous cycle: implications for the gametes-oviduct interactions and the environment of the potential embryo. *BMC Genomics* **19**, 622, doi:10.1186/s12864-018-4982-5 (2018).

6 Kang, J. *et al.* Ribosomal proteins and human diseases: molecular mechanisms and targeted therapy. *Signal Transduct Target Ther* **6**, 323, doi:10.1038/s41392-021-00728-8 (2021).

7 James, N. E. *et al.* Septin-2 is overexpressed in epithelial ovarian cancer and mediates proliferation via regulation of cellular metabolic proteins. *Oncotarget* **10**, 2959-2972, doi:10.18632/oncotarget.26836 (2019).

8 Santana-Rivera, Y. *et al.* Reduced expression of enolase-1 correlates with high intracellular glucose levels and increased senescence in cisplatin-resistant ovarian cancer cells. *Am J Transl Res* **12**, 1275-1292 (2020).

9 Sohn, M. H. *et al.* Classification of High-Grade Serous Ovarian Carcinoma by Epithelial-to-Mesenchymal Transition Signature and Homologous Recombination Repair Genes. *Genes (Basel)* **12**, doi:10.3390/genes12071103 (2021).

10 Zhou, Y. *et al.* Metascape provides a biologist-oriented resource for the analysis of systems-level datasets. *Nat Commun* **10**, 1523, doi:10.1038/s41467-019-09234-6 (2019).
