# Supplementary material for: Lineage specific extracellular vesicle-associated protein biomarkers for the early detection of high grade serous ovarian cancer
Source: Sci Rep. 2023 Oct 26;13:18341. doi: 10.1038/s41598-023-44050-5 (PMC10603107; doi:10.1038/s41598-023-44050-5)
Supplement: Supplementary file 6 — Supplementary Information 6. [file 41598_2023_44050_MOESM6_ESM.pdf]

Description of supplementary files, figures and tables.

<u>**Supplementary Figures**</u>

Supplementary Figure 1. NTA data of EVs isolated from primary ovarian tumor tissue explants at 24 h and 48 h.

Supplementary Figure 2. NTA particle counts and total EV protein data.

Supplementary Figure 3. Characterization of cell line EVs.

Supplementary Figure 4. Heatmaps of the tetraspanin distribution using SP-IRIS and fluorescence.

Supplementary Figure 5. Heatmap of FT/HGSOC-associated EV proteins identified by mass spectrometry which have been previously reported in the literature as serum-associated biomarkers for ovarian cancer.

Supplementary Figure 6. Full length unprocessed capillary western blots.

Supplementary Figure 7. Violin plots show the distribution of IHC expression scores (H-score) from healthy FT, primary HGSOC, and metastatic tissue samples.

Supplementary Figure 8. Gene ontology analysis of FT/HGSOC core proteome.

Supplementary Figure 9. Comparison of FT/HGSOC core proteome to Vesiclepedia database.

Supplementary Figure 10. StringDB analysis of 52 unique proteins from FT/HGSOC core proteome.

Supplementary Figure 11. Metascape analysis of 324 unique proteins in HGSOC samples.

Supplementary Tables

Supplementary Table 1. List of 75 transmembrane proteins ranked according to fold change.

Supplementary Table 2. Area under the curve values for early and late stage ovarian cancer patient samples compared to healthy controls on the ExoProfile chip.

Supplementary Table 3. Statistical analysis for each exo-protein biomarker on the ExoProfile chip for the 10 ovarian cancer and 20 healthy controls plasma within the specificity sample set.

Supplementary Table 4. Antibodies used in this study.

Supplementary Table 5. List of monotone and non-monotone markers.

Supplementary Table 6. List of 324 unique proteins in HGSOC samples.

Supplementary Files

Supplementary File 1. Clinical information of patients included in this study.

Supplementary File 2. List of 985 FT/HGSOC proteins including statistical analysis using edgeR and qprot values.

Supplementary File 3. Bioinformatic analysis of FT/HGSOC identified via LC-MS/MS.

Supplementary File 4. List of SwissProt reviewed transmembrane proteins acquired July 2021.

Supplementary File 5. Gene ontology analysis of FT/HGSOC core proteome.

Supplementary Figure 1.

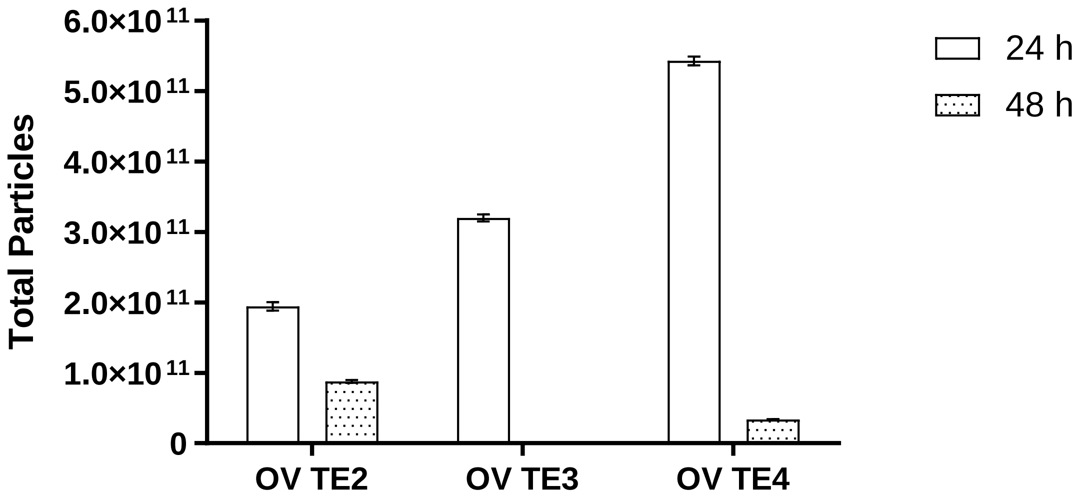

Supplementary Figure 1. NTA data of EVs isolated from primary ovarian tumor tissue explants at 24 h and 48 h. p-values were calculated using Mann-Whitney U test and error bars indicate mean with s.e.m.

Supplementary Figure 2.

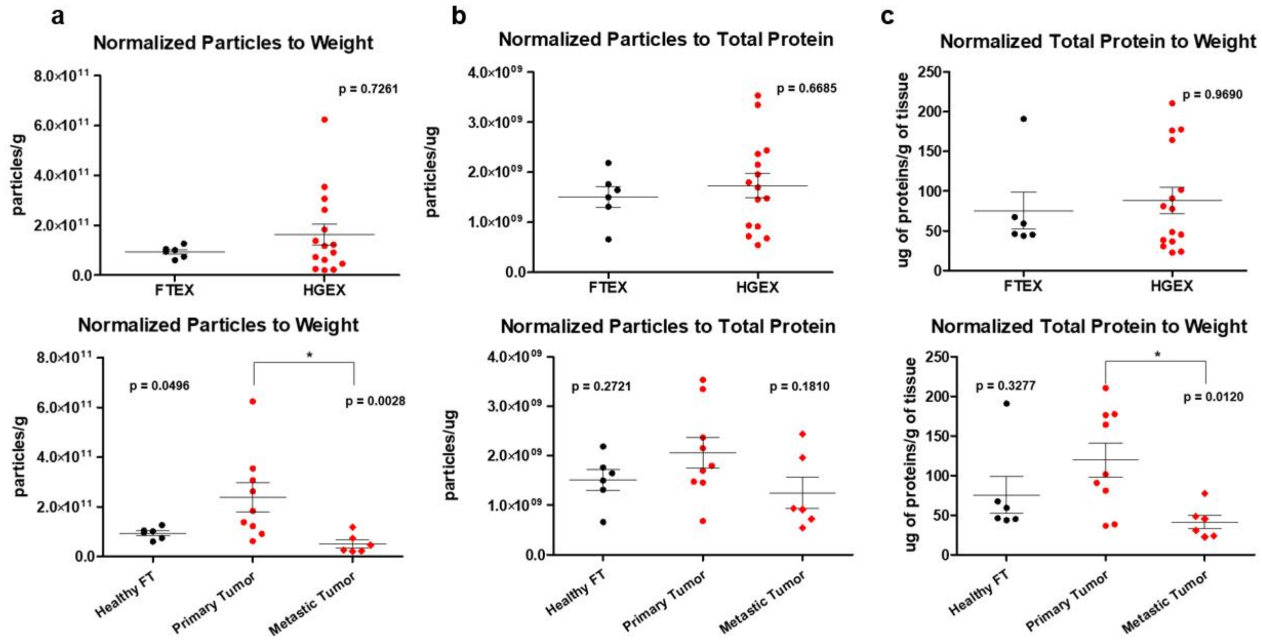

Supplementary Figure 2. NTA particle counts and total EV protein data. NTA data were normalized to **a**) weight of tissue explant (healthy FT or HGSOC) or **b**) total EV protein. **c**) Total EV protein normalized to weight of tissue explant. These data show that FT and HGSOC tissue explants generated similar EV yields. p-values were calculated by comparing primary tumor with healthy FT or metastatic tumor using Mann-Whitney U test. Asterisks indicates $p<0.05$ or significance. Error bars show mean with s.e.m.

Supplementary Figure 3.

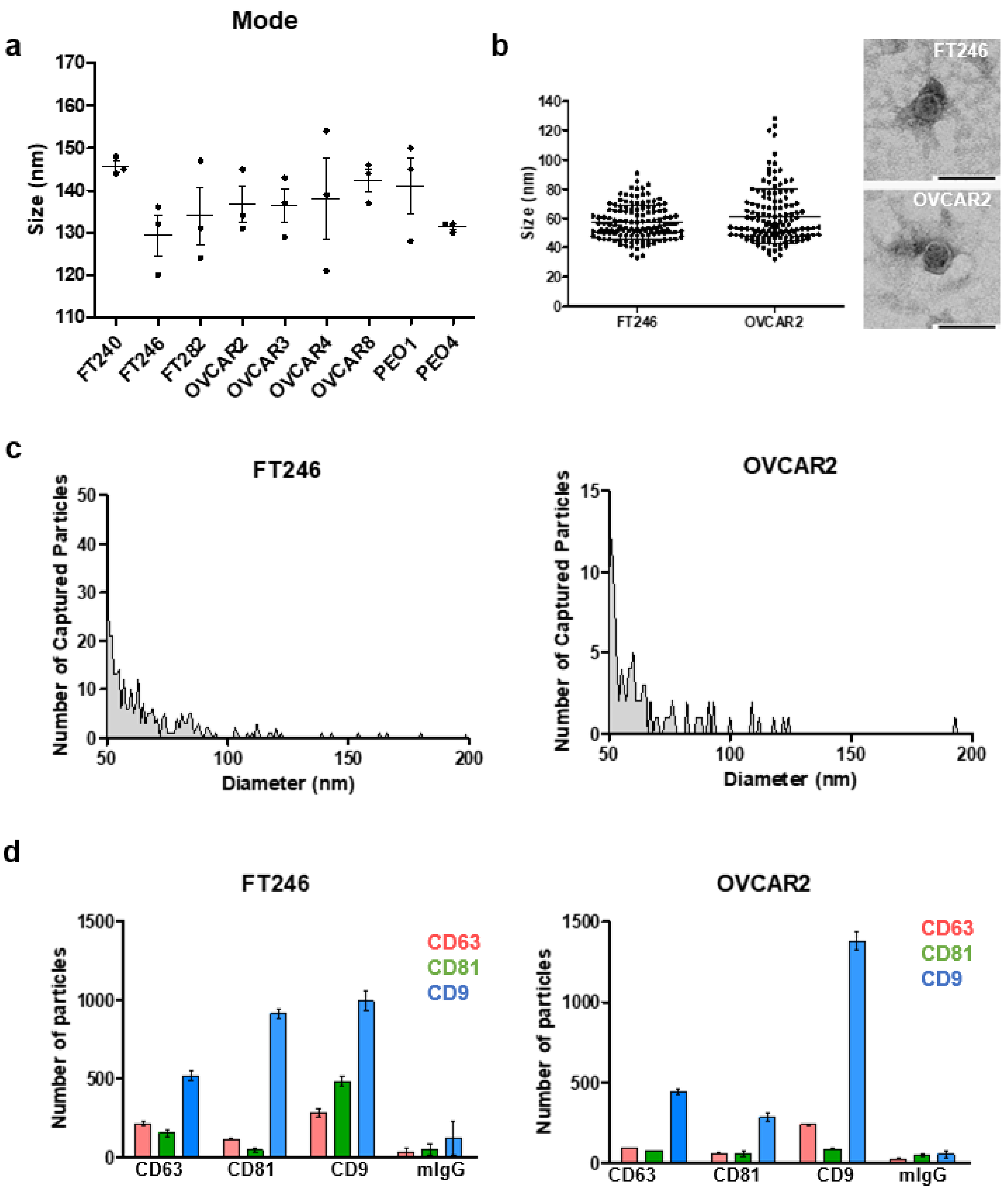

Supplementary Figure 3. Characterization of cell line EVs. a) Mode size of EVs via NTA. Error bars show mean particle size with s.e.m. **b)** EV particles were imaged for representative samples by TEM (n=130) at x30K magnification. p-values were calculated using Mann-Whitney U test and error bars indicate mean particle size with s.e.m. **c)** Size

distribution of SP-IRIS (anti-CD81 capture). **d)** Tetraspanin profile of cell line derived EVs captured using commonly expressed EV tetraspanins: CD9, CD63 and CD81, and probed with detection antibodies conjugated to Alexa Fluor dyes: CD9-AF488 (blue), CD63-AF647 (pink) and CD81-AF555 (green). The error bars represent the mean particle count with s.e.m.

Supplementary Figure 4.

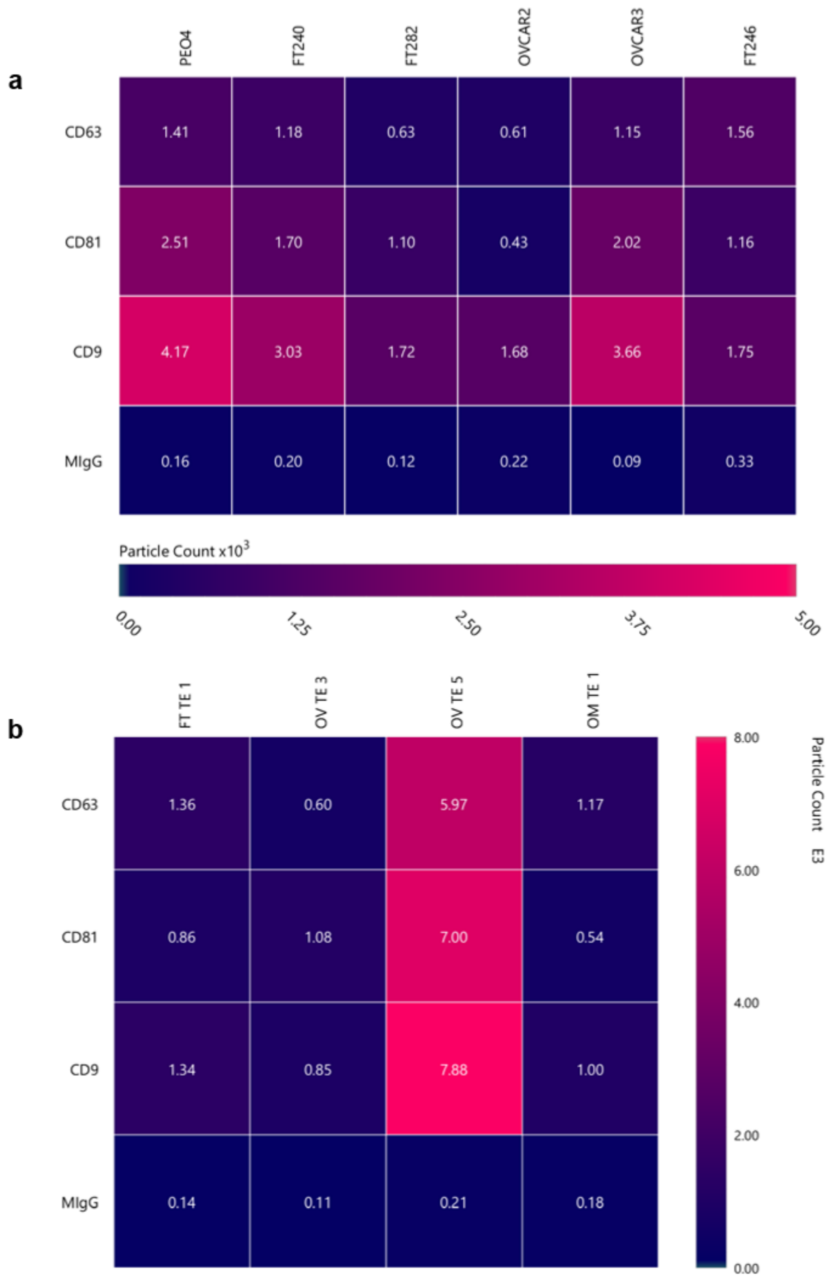

Supplementary Figure 4. Heatmaps of the tetraspanin distribution using SP-IRIS and fluorescence. a) Cell line EVs, and **b)** tissue derived EVs. The values in each box represent the actual number of EVs captured and quantified on the chip using fluorescence.

Supplementary Figure 5.

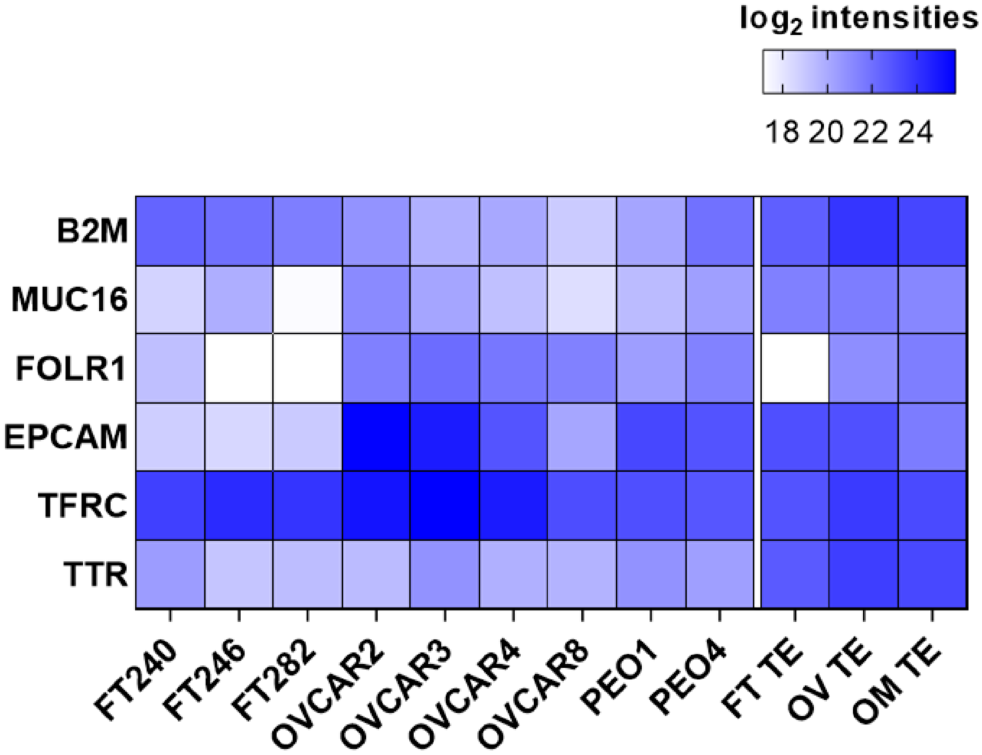

Supplementary Figure 5. Heatmap of FT/HGSOC-associated EV proteins identified by mass spectrometry. Shown are proteins that have been previously reported in the literature as serum-associated biomarkers for ovarian cancer.

Supplementary Figure 6.

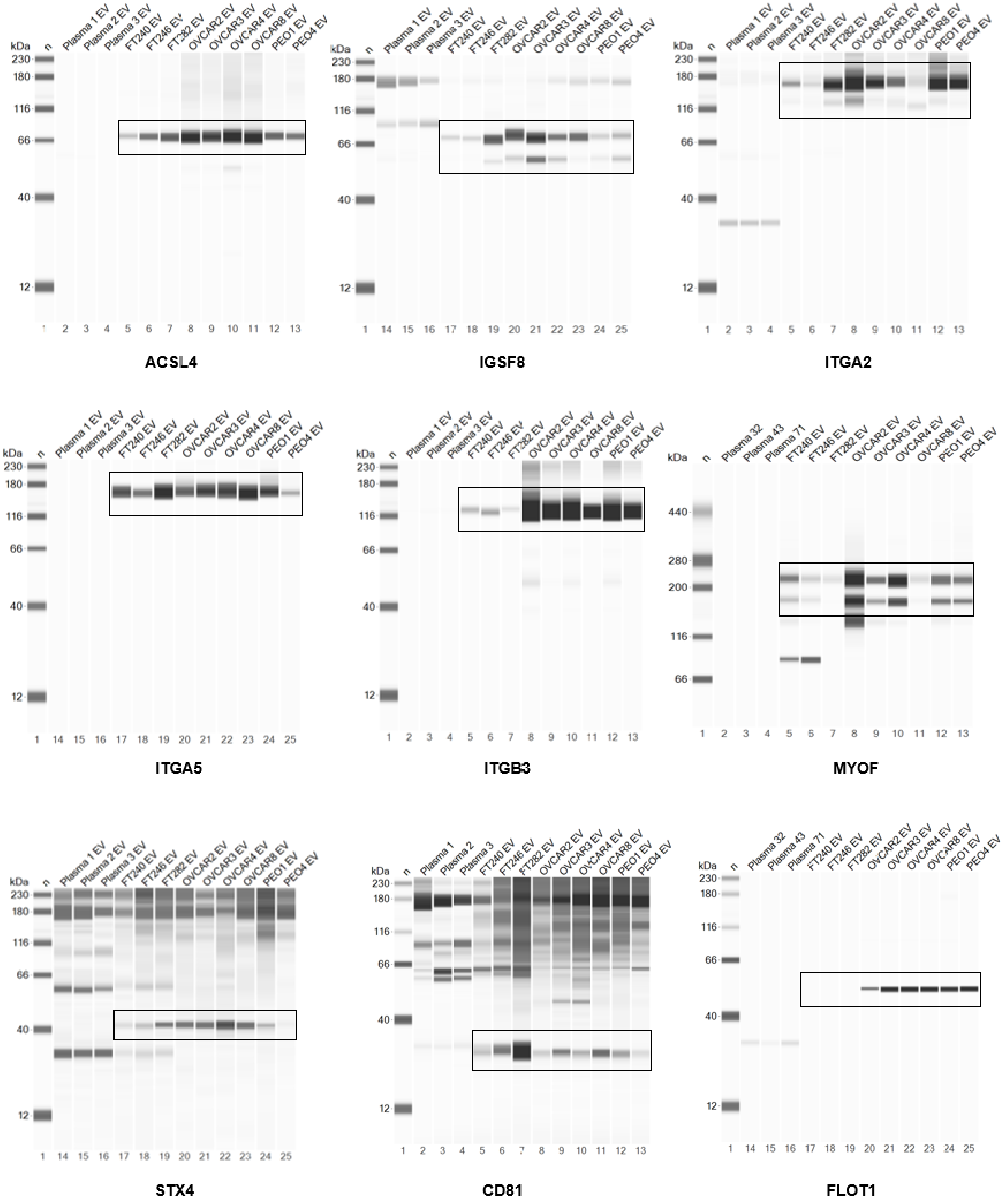

Supplementary Figure 6. Full length unprocessed capillary western blots.

Supplementary Figure 7.

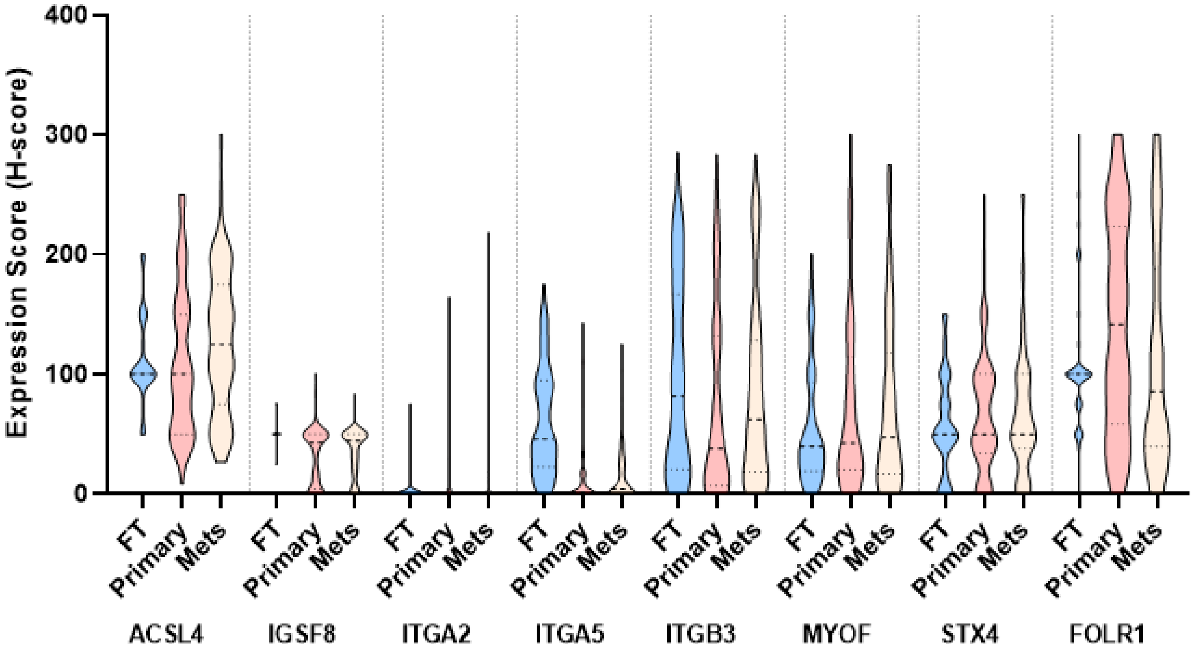

Supplementary Figure 7. Violin plots show the distribution of IHC expression scores (H-score) from healthy FT, primary HGSOC, and metastatic tissue samples. All tissue cores were scored by a pathologist. The medians are represented with dashed lines, and the dotted lines represent quartiles.

Supplementary Figure 8.

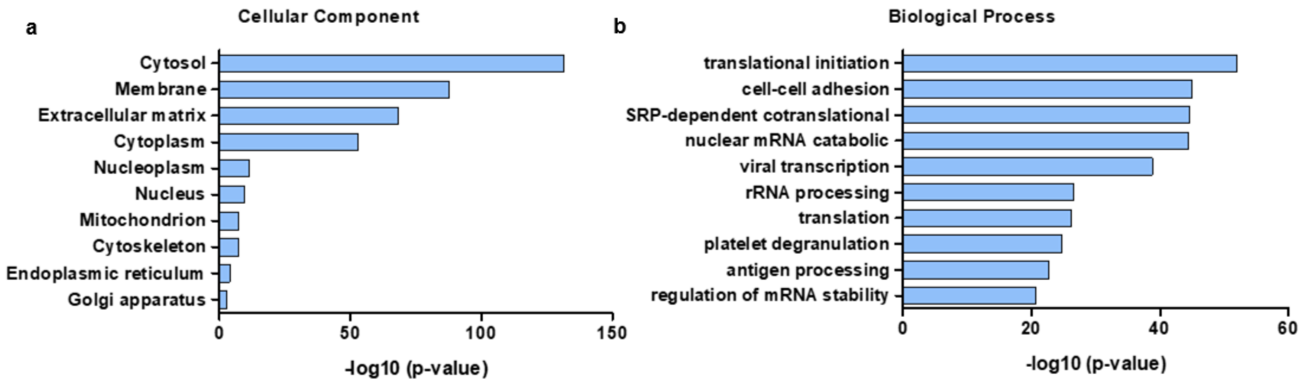

Supplementary Figure 8. Gene ontology analysis of FT/HGSOC core proteome. Shown are **a)** cellular component and **b)** biological process.

Supplementary Figure 9.

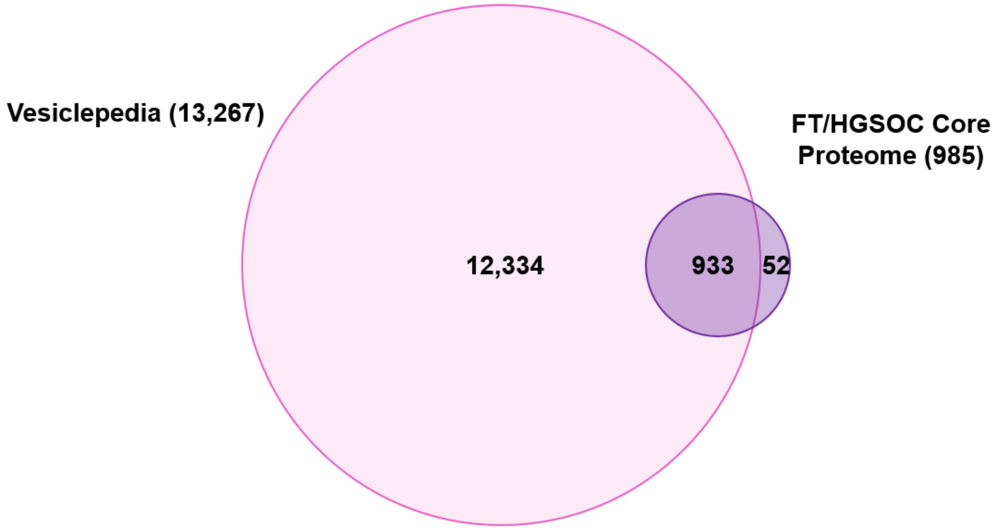

Supplementary Figure 9. Comparison of FT/HGSOC core proteome to Vesiclepedia database.

Supplementary Figure 10.

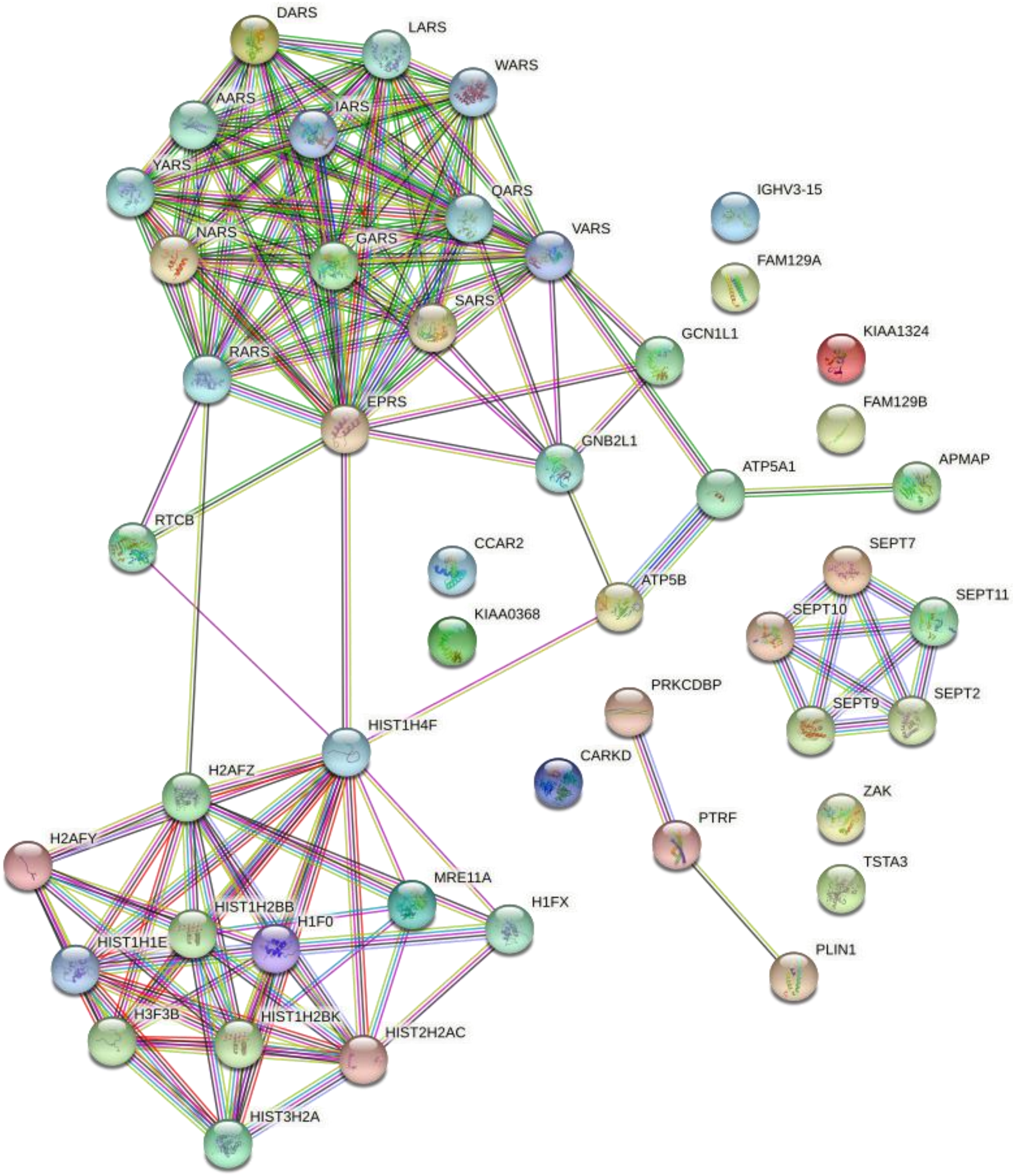

Supplementary Figure 10. StringDB of the 52 unique proteins from FT/HGSOC core proteome that were not found in Vesiclepedia database.

Supplemental Figure 11.

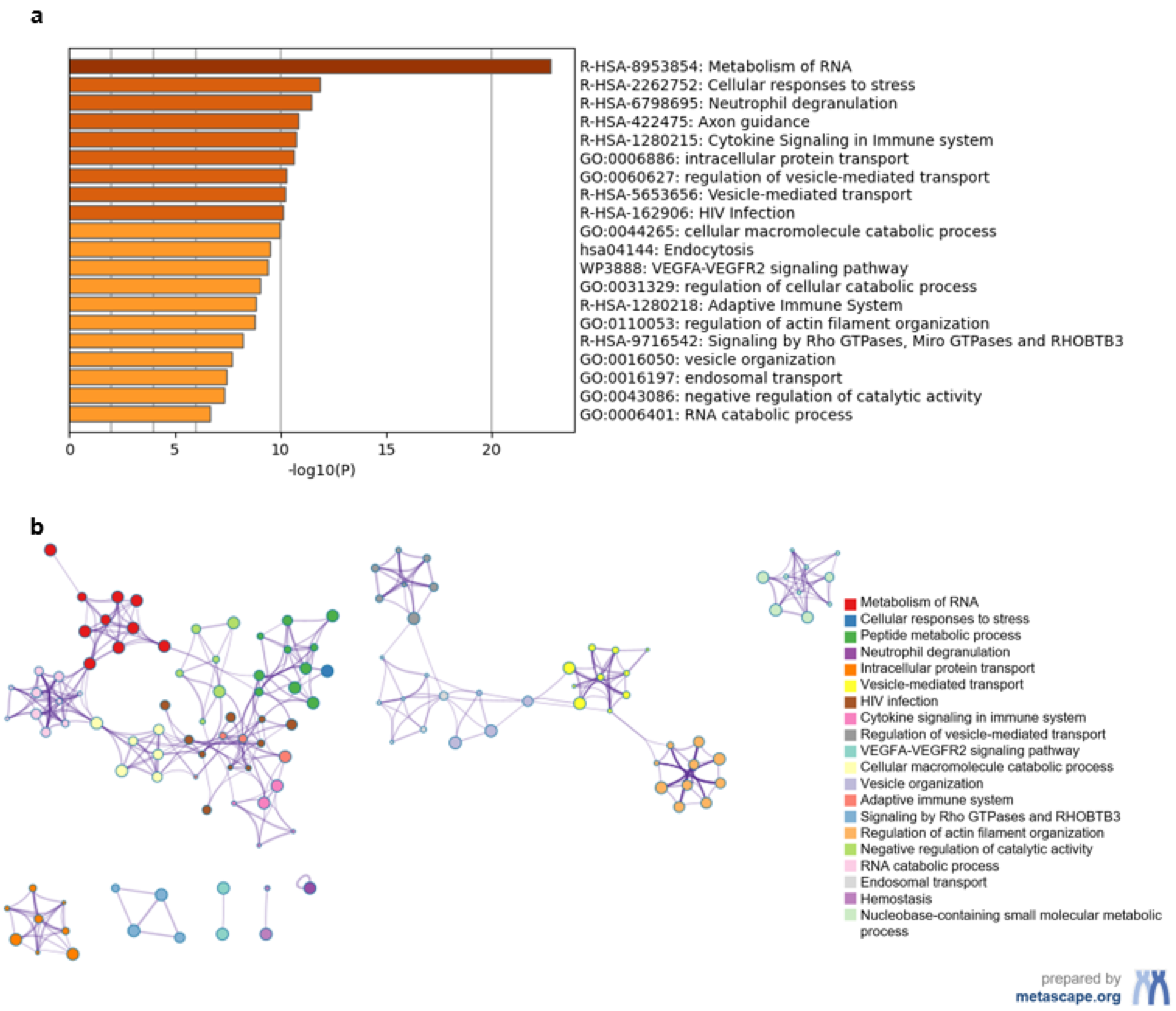

Supplementary Figure 11. Metascape analysis of 324 unique proteins in HGSOC samples. a) Statistically enriched terms based on GO/KEGG and canonical pathways and **b)** network layout of a subset of representative terms. Each term is represented by a circular node.
