# Supplementary material for: Lineage specific extracellular vesicle-associated protein biomarkers for the early detection of high grade serous ovarian cancer
Source: Sci Rep. 2023 Oct 26;13:18341. doi: 10.1038/s41598-023-44050-5 (PMC10603107; doi:10.1038/s41598-023-44050-5)
Supplement: Supplementary file 7 — Supplementary Tables. [file 41598_2023_44050_MOESM7_ESM.pdf]

Supplementary Table 1

75 transmembrane proteins found in FT/HGSOC core proteome; ranked according to fold change
9 proteins were excluded because they are considered common EV proteins (highlighted in blue, see text for details)
46 proteins selected for further analysis are highlighted in gold.
IGHM and ADAM10 were excluded (highlighted in red, see text for details)

UniProt ID	Gene ID	Detected in top 100 Common EV proteins in ExoCarta/Vesiclepedia	log2 FC (HGSOC/FT)	-log10 (p value)	Detected in Reference # 53
P07099	EPHX1	No	1.35	1.25	No
P16422	EPCAM	No	1.30	0.75	Yes
P05106	ITGB3	No	1.22	0.76	No
P15151	PVR	No	1.16	1.53	Yes
P35613	BSG	Yes	1.07	0.44	NA
Q92896	GLG1	No	0.80	0.89	Yes
P23229	ITGA6	Yes	0.74	0.69	NA
O00299	CLIC1	Yes	0.73	1.20	NA
Q9P2B2	PTGFRN	Yes	0.73	0.66	NA
P02730	SLC4A1	No	0.68	0.41	Yes
P08648	ITGA5	No	0.61	0.27	Yes
Q9UBG0	MRC2	No	0.59	0.41	Yes
Q96NY7	CLIC6	No	0.54	0.35	No
O00468	AGRN	No	0.53	0.36	No
P15144	ANPEP	No	0.48	0.38	Yes
P08514	ITGA2B	No	0.42	0.18	Yes
O43490	PROM1	No	0.35	0.21	Yes
P16435	POR	No	0.33	0.46	No
Q9BSJ8	ESYT1	No	0.32	0.30	No
P00533	EGFR	No	0.13	0.06	Yes
P08575	PTPRC	No	0.11	0.06	Yes
O15031	PLXNB2	No	0.11	0.09	Yes
Q5T4S7	UBR4	No	0.11	0.11	No
P17301	ITGA2	No	0.09	0.04	Yes
O60488	ACSL4	No	0.09	0.05	No

Q16853	AOC3	No	0.07	0.03	Yes
Q07954	LRP1	No	0.05	0.03	Yes
P01871	IGHM	No	-0.02	0.01	Yes
P11279	LAMP1	No	-0.03	0.01	No
P05187	ALPP	No	-0.05	0.02	No
Q12797	ASPH	No	-0.05	0.03	No
Q12846	STX4	No	-0.05	0.05	Yes
Q13308	PTK7	No	-0.07	0.04	Yes
Q969P0	IGSF8	No	-0.07	0.03	Yes
P33121	ACSL1	No	-0.07	0.03	No
P05023	ATP1A1	Yes	-0.08	0.04	NA
O00264	PGRMC1	No	-0.10	0.07	No
Q6DD88	ATL3	No	-0.10	0.06	No
P20020	ATP2B1	No	-0.19	0.14	Yes
P49755	TMED10	No	-0.19	0.18	No
O14672	ADAM10	No	-0.22	0.11	Yes
P23634	ATP2B4	No	-0.27	0.30	Yes
Q10589	BST2	No	-0.29	0.18	Yes
P22897	MRC1	No	-0.29	0.24	No
Q9NZM1	MYOF	No	-0.30	0.32	Yes
P51572	BCAP31	No	-0.31	0.37	No
P08069	IGF1R	No	-0.33	0.20	Yes
P05141	SLC25A5	No	-0.34	0.41	No
P02786	TFRC	Yes	-0.40	0.21	NA
P36269	GGT5	No	-0.43	0.49	Yes
Q9HDC9	APMAP	No	-0.44	0.50	Yes
Q13740	ALCAM	No	-0.45	0.78	Yes
P08195	SLC3A2	Yes	-0.48	0.22	NA
Q6UVK1	CSPG4	No	-0.49	0.78	Yes
O00592	PODXL	No	-0.60	0.63	NA
P11717	IGF2R	No	-0.62	0.27	NA
Q6UXG2	ELAPOR1	No	-0.66	0.45	NA
P06756	ITGAV	No	-0.70	0.69	NA
Q9Y696	CLIC4	No	-0.72	1.02	NA

Q08722	CD47	No	-0.77	1.12	NA
P50851	LRBA	No	-0.77	1.35	NA
P49257	LMAN1	No	-0.79	1.31	NA
P07357	C8A	No	-0.81	0.95	NA
P16615	ATP2A2	No	-0.85	2.45	NA
P27824	CANX	No	-0.87	2.31	NA
P54289	CACNA2D1	No	-0.98	1.25	NA
P04843	RPN1	No	-1.00	1.25	NA
Q16647	PTGIS	No	-1.16	1.25	NA
Q9Y639	NPTN	No	-1.23	2.12	NA
P21926	CD9	Yes	-1.39	1.52	NA
P02748	C9	No	-1.48	2.31	NA
P05556	ITGB1	Yes	-1.53	1.67	NA
P18084	ITGB5	No	-1.78	3.23	NA
P26006	ITGA3	No	-1.89	2.26	NA
P29317	EPHA2	No	-2.09	2.10	NA

Supplementary Table 2

Area under the curve values for early stage and late stage ovarian cancer (OC) patient samples compared to healthy controls (HC) on the ExoProfile chip.

Exo-Protein	HC vs Early Stage OC AUC	HC vs Late Stage OC AUC
IGSF8	0.820	0.970
ITGA2	0.830	0.940
MYOF	0.770	0.930
ACSL4	0.920	0.910
ITGB3	1.000	0.960
ITGA5	0.970	0.930
FOLR1	0.970	0.880

Supplementary Table 3

Statistical analysis for each exo-protein biomarker on the ExoProfile chip for the 10 ovarian cancer and 20 healthy controls plasma within the specificity sample set.

Exo-Protein	AUC	Standard	CI_lower	CI_upper	p-value-2t	Sensitivity at 99.8% Specificity	PPV
**IGSF8**	0.985	0.018	0.949	1	1.09×10^{-4}	0.7	0.123
**ITGA2**	1	NA	NA	NA	6.00×10^{-6}	1	0.167
**MYOF**	0.98	0.023	0.935	1	1.33×10^{-5}	0.6	0.107
**ACSL4**	0.995	0.009	0.978	1	7.34×10^{-6}	0.9	0.153
**ITGB3**	0.985	0.018	0.95	1	1.09×10^{-5}	0.9	0.153
**ITGA5**	0.99	0.013	0.964	1	8.96×10^{-6}	0.9	0.153
**FOLR1**	0.995	0.009	0.978	1	7.34×10^{-6}	0.9	0.153
**Linear Combination of IGSF8 & ITGA5**	0.995	0.009	0.978	1	7.34×10^{-6}	0.9	0.138

Supplementary Table 4

Antibodies used in this study.

Antibodies	Manufacturer	Catalog #	Dilution	Application
ACSL4	Novus Biologicals	NBP2-16401	1:50	Wes
			1:200	IHC
ACSL4	Novus Biologicals	NB300-861	1:500	ExoProfile chip
CA-125	Abcam	ab274402	NA	ELISA
CD81	Proteintech	66866-1-Ig	1:100	Wes
CD81	Ancell	302-820	1:5	ExoProfile chip
Flotillin-1	Santa Cruz	sc-74566	1:50	Wes
FOLR1	R&D systems	AF5646	1:500	ExoProfile chip
FOLR1	Invitrogen	PA5-24186	1:400	IHC
IGSF8	Novus Biologicals	AF3117	1:20	Wes
IGSF8	R&D systems	MAB31171-100	1:100	ExoProfile chip
IGSF8	Invitrogen	PA5-52875	1:100	IHC
ITGA2	Novus Biologicals	NBP2-76483	1:20	Wes
			1:200	IHC
ITGA2	Novus Biologicals	NBP3-03851	1:100	ExoProfile chip
ITGA5	Novus Biologicals	NBP1-84576	1:50	Wes
			1:200	IHC
ITGA5	R&D systems	AF1864	1:500	ExoProfile chip
ITGB3	Novus Biologicals	NBP2-67416	1:50	Wes
			1:50	IHC
ITGB3	R&D systems	AF2266	1:500	ExoProfile chip
MYOF	Novus Biologicals	NBP1-84694	1:20	Wes
			1:100	ExoProfile chip
			1:200	IHC
p53	Dako	IR616	NA	IHC
STX4	Novus Biologicals	MAB7894-SP	1:20	Wes
STX4	Invitrogen	PA5-51560	1:300	IHC
Donkey anti-Goat IgG (H+L) secondary antibody (biotin)	Novus Biologicals	NBP1-74820	1:500	ExoProfile chip
Donkey anti-Rabbit IgG (H+L) secondary antibody (biotin)	Novus Biologicals	NBP1-75288	1:500	ExoProfile chip

Supplementary Table 5

List of monotone markers.

Accession	Gene Name	AUC	p-value	|AUC-0.5|+0.5	logFC
P63010	AP2B1	0.04233	0.00010	0.95767	-1.68695
P23528	CFL1	0.10053	0.00069	0.89947	-1.87569
P63261	ACTG1	0.12169	0.00131	0.87831	-2.68205
P11940	PABPC1	0.12169	0.00131	0.87831	-1.92556
P31946	YWHAB	0.13228	0.00179	0.86772	-1.14743
P29692	EEF1D	0.14286	0.00243	0.85714	-2.72388
P30481	HLA-B	0.14286	0.00243	0.85714	-1.69459
Q99439	CNN2	0.14286	0.00243	0.85714	-2.03771
Q00341	HDLBP	0.14815	0.00282	0.85185	-1.20565
P02649	APOE	0.15344	0.00326	0.84656	-1.33752
P61981	YWHAG	0.15873	0.00377	0.84127	-1.25107
P20339	RAB5A	0.15873	0.00377	0.84127	-1.29313
P51148	RAB5C	0.16931	0.00502	0.83069	-1.49546
P40261	NNMT	0.17460	0.00577	0.82540	-2.65944
Q15582	TGFBI	0.17989	0.00662	0.82011	-2.18810
Q99829	CPNE1	0.18519	0.00758	0.81481	-1.99417
P63313	TMSB10	0.18519	0.00758	0.81481	-1.27912
P26006	ITGA3	0.82011	0.00662	0.82011	1.34115
P18084	ITGB5	0.82540	0.00577	0.82540	2.56944
P04216	THY1	0.83069	0.00502	0.83069	1.32160
P02768	ALB	0.85185	0.00282	0.85185	1.04041
Q6UXG2	ELAPOR1	0.85185	0.00282	0.85185	-0.60241
Q8TE73	DNAH5	0.86243	0.00209	0.86243	1.62714
Q9Y639	NPTN	0.87302	0.00154	0.87302	1.63668
P04217	A1BG	0.88889	0.00095	0.88889	1.26477
Q99798	ACO2	0.93651	0.00021	0.93651	1.29714
O43854	EDIL3	0.98413	0.00004	0.98413	4.69203

List of non-monotone markers

Accession	Gene Name	AUC	p-value	|AUC-0.5|+0.5	logFC
P68366	TUBA4A	0.62434	0.29792	0.62434	1.79590
P46781	RPS9	0.63492	0.25788	0.63492	1.24112
Q9NTK5	OLA1	0.40212	0.41529	0.59788	-0.73955
P31943	HNRNPH1	0.45503	0.71731	0.54497	-0.81772
P34913	EPHX2	0.59788	0.41529	0.59788	0.52877
Q06787	FMR1	0.58201	0.49724	0.58201	-0.05896
O43866	CD5L	0.58201	0.49724	0.58201	0.42504
P61769	B2M	0.45503	0.71731	0.54497	-0.94536
P61158	ACTR3	0.56085	0.61861	0.56085	0.35186
Q9BTU6	PI4K2A	0.50265	1.00000	0.50265	1.49655
P00338	LDHA	0.42328	0.52635	0.57672	-1.21965
Q15008	PSMD6	0.37566	0.29792	0.62434	-0.27057
P62280	RPS11	0.62434	0.29792	0.62434	0.88494
P62191	PSMC1	0.37037	0.27741	0.62963	-0.36233
P50990	CCT8	0.53968	0.75140	0.53968	0.11108
Q92878	RAD50	0.49735	1.00000	0.50265	-0.35020

Supplementary Table 6

List of 324 unique proteins in HGSOC samples.

UniProt ID	Gene Name	UniProt ID	Gene Name	UniProt ID	Gene Name	UniProt ID	Gene Name
P63267	ACTG2	Q09028	RBBP4	Q96F85	CNRIP1	Q9H269	VPS16
P00739	HPR	P01824	IGHV4-39	P05771	PRKCB	Q9NR50	EIF2B3
Q5QNW6	H2BC18	Q13547	HDAC1	Q6IAA8	LAMTOR1	P55011	SLC12A2
Q9BTM1	H2AJ	Q10567	AP1B1	Q9UI12	ATP6V1H	Q96M27	PRRC1
P05455	SSB	P62841	RPS15	P09661	SNRPA1	P26368	U2AF2
P02042	HBD	P60228	EIF3E	Q92817	EVPL	Q13188	STK3
P16104	H2AX	P30044	PRDX5	Q6IBS0	TWF2	O43813	LANCL1
Q8IUE6	H2AC21	O75165	DNAJC13	P46976	GYG1	P13693	TPT1
P28161	GSTM2	O75351	VPS4B	P55769	SNU13	O95490	ADGRL2
Q7Z6Z7	HUWE1	Q92743	HTRA1	P20160	AZU1	Q9NVP1	DDX18
P53618	COPB1	P16284	PECAM1	Q12972	PPP1R8	O43148	RNMT
Q15366	PCBP2	Q15485	FCN2	P08621	SNRNP70	P50502	ST13
P32969	RPL9	A0A0B4J1X5	IGHV3-74	Q92485	SMPDL3B	P30084	ECHS1
P53675	CLTCL1	Q9NQW7	XPNPEP1	Q9Y6E0	STK24	Q05655	PRKCD
Q9ULA0	DNPEP	O94776	MTA2	Q6YP21	KYAT3	Q9BVG4	PBDC1
Q12906	ILF3	Q9H3U1	UNC45A	P36405	ARL3	P29590	PML
Q14103	HNRNPD	P47755	CAPZA2	P36404	ARL2	Q9GZZ1	NAA50
P30043	BLVRB	Q86WI1	PKHD1L1	P01817	IGHV2-5	P61764	STXBP1
Q07065	CKAP4	Q6XQN6	NAPRT	Q14376	GALE	P46926	GNPDA1
P55795	HNRNPH2	P20851	C4BPB	P12821	ACE	P60983	GMFB
P11217	PYGM	Q9BWD1	ACAT2	P13797	PLS3	O60825	PFKFB2
P40763	STAT3	O95671	ASMTL	P06702	S100A9	P49207	RPL34
Q9NQR4	NIT2	P41226	UBA7	Q8TAT6	NPLOC4	Q99816	TSG101
P01833	PIGR	Q8WVM8	SCFD1	O76003	GLRX3	P61923	COPZ1
O43865	AHCYL1	P26196	DDX6	Q15185	PTGES3	Q13107	USP4
P27695	APEX1	P42229	STAT5A	Q9UI30	TRMT112	Q9P0K7	RAI14
P67809	YBX1	O14828	SCAMP3	P60866	RPS20	P10599	TXN
O94874	UFL1	Q15477	SKIV2L	Q9UHG3	PCYOX1	O75915	ARL6IP5
Q9Y285	FARSA	P23921	RRM1	P13796	LCP1	P07093	SERPINE2
Q9UBQ0	VPS29	Q02880	TOP2B	P45880	VDAC2	Q9NZL9	MAT2B
P31689	DNAJA1	P06730	EIF4E	Q5VW32	BROX	Q9UEY8	ADD3

Q9Y5B9	SUPT16H	Q15042	RAB3GAP1	Q15370	ELOB	Q96JH7	VCPIP1
P19971	TYMP	P40121	CAPG	Q16787	LAMA3	O60493	SNX3
P51888	PRELP	Q7Z4W1	DCXR	A0A087WW87	IGKV2-40	P00451	F8
Q07960	ARHGAP1	P62314	SNRPD1	P09972	ALDOC	Q9BS26	ERP44
P62140	PPP1CB	P60891	PRPS1	P09758	TACSTD2	Q15813	TBCE
P26599	PTBP1	P61201	COPS2	O15247	CLIC2	P04080	CSTB
Q9P258	RCC2	A0A0C4DH38	IGHV5-51	P62316	SNRPD2	A0A075B6J9	IGLV2-18
P08243	ASNS	P16050	ALOX15	Q7L1Q6	BZW1	Q9Y333	LSM2
Q7Z406	MYH14	O94903	PLPBP	P26447	S100A4	P62942	FKBP1A
P36543	ATP6V1E1	P09496	CLTA	Q8WXX5	DNAJC9	P00390	GSR
P30876	POLR2B	Q9HAV0	GNB4	Q3KQV9	UAP1L1	Q01581	HMGCS1
Q14914	PTGR1	P78386	KRT85	Q13464	ROCK1	P55039	DRG2
P09874	PARP1	P52943	CRIP2	Q9UKD2	MRTO4	Q9BTT0	ANP32E
Q96KP4	CNDP2	Q9UL46	PSME2	Q02252	ALDH6A1	Q14232	EIF2B1
O15372	EIF3H	P09104	ENO2	Q9BXR0	QTRT1	Q8N684	CPSF7
P26639	TARS1	Q99707	MTR	Q8NCA5	FAM98A	Q8WW22	DNAJA4
P51570	GALK1	Q9HB71	CACYBP	Q13177	PAK2	P62312	LSM6
Q15369	ELOC	O75832	PSMD10	Q9UBW5	BIN2	P61326	MAGOH
O15143	ARPC1B	Q14160	SCRIB	P42226	STAT6	P59665	DEFA1
P57737	CORO7	P02654	APOC1	P62318	SNRPD3	O00743	PPP6C
O43396	TXNL1	Q9UKG1	APPL1	Q13033	STRN3	O60343	TBC1D4
O00203	AP3B1	Q8N6C5	IGSF1	O00187	MASP2	Q13404	UBE2V1
O96019	ACTL6A	Q9NZA1	CLIC5	O95352	ATG7	O43291	SPINT2
O43681	GET3	Q96H79	ZC3HAV1L	Q99575	POP1	P06454	PTMA
P50552	VASP	P40925	MDH1	P05362	ICAM1	Q9UNE7	STUB1
Q93009	USP7	P21281	ATP6V1B2	Q9Y2Q3	GSTK1	Q8N6H7	ARFGAP2
P31150	GDI1	P09471	GNAO1	P35052	GPC1	Q14767	LTBP2
P49721	PSMB2	P08240	SRPRA	P06276	BCHE	P98172	EFNB1
Q9NR99	MXRA5	P24928	POLR2A	P04632	CAPNS1	Q8WUW1	BRK1
P61221	ABCE1	P41240	CSK	Q8WXH0	SYNE2	Q92979	EMG1
O95865	DDAH2	P05107	ITGB2	O00194	RAB27B	O43617	TRAPPC3
Q13363	CTBP1	P22061	PCMT1	P43487	RANBP1	Q8TDY4	ASAP3
P80511	S100A12	P11388	TOP2A	P48556	PSMD8	P51151	RAB9A
P53999	SUB1	P05186	ALPL	Q9Y224	RTRAF	Q07021	C1QBP

P35270	SPR	Q14694	USP10	Q8N8N7	PTGR2	Q5HYI8	RABL3
Q9NRW1	RAB6B	O95810	CAVIN2	Q04323	UBXN1	Q6VY07	PACS1
P52907	CAPZA1	Q9Y6K5	OAS3	P48426	PIP4K2A	O95239	KIF4A
P68402	PAFAH1B2	Q96QR8	PURB	P18031	PTPN1	Q96SB4	SRPK1
P30491	HLA-B	Q99447	PCYT2	Q9NP79	VTA1	Q969T7	NT5C3B
P11277	SPTB	O15400	STX7	Q92973	TNPO1	O00221	NFKBIE
P11215	ITGAM	Q9H3S7	PTPN23	Q9UL26	RAB22A	Q14444	CAPRIN1
P52788	SMS	P56199	ITGA1	Q8TEQ6	GEMIN5	P16112	ACAN
P52597	HNRNPF	P53367	ARFIP1	P35637	FUS	O14920	IKBKB
Q15046	KARS1	Q9NRW7	VPS45	P62714	PPP2CB	Q6ICL3	TANGO2
P30085	CMPK1	Q9BQS8	FYCO1	O95372	LYPLA2	Q5T7R7	C1orf185
P25205	MCM3	P20839	IMPDH1	Q2NL82	TSR1	P07477	PRSS1
P62829	RPL23	O94832	MYO1D	P18077	RPL35A	P10915	HAPLN1
P51610	HCFC1	P61026	RAB10	Q9Y624	F11R	Q14451	GRB7
P62888	RPL30	Q06203	PPAT	Q96S55	WRNIP1	Q5T8A7	PPP1R26
Q8NE71	ABCF1	Q6PGP7	TTC37	P15328	FOLR1	Q6ZVH7	ESPNL
